# *NKX2*-*1-AS1* negatively regulates *CD274*/PD-L1, cell-cell interaction genes, and limits human lung carcinoma cell migration

**DOI:** 10.1038/s41598-018-32793-5

**Published:** 2018-09-26

**Authors:** Hasmeena Kathuria, Guetchyn Millien, Liam McNally, Adam C. Gower, Jean-Bosco Tagne, Yuxia Cao, Maria I. Ramirez

**Affiliations:** 1 The Pulmonary Center, Boston University School of Medicine, 72 E. Concord St, Boston, MA 02118 USA; 2 Clinical and Translational Science Institute, Boston University School of Medicine, 72 E. Concord St, Boston, MA 02118 USA; 3 Department of Pathology and Laboratory Medicine, Boston University School of Medicine, 72 E. Concord St, Boston, MA 02118 USA; 4 Present Address: Center for Translational Medicine, Department of Medicine, Sidney Kimmel Medical College, Thomas Jefferson University, 1020 Locust St, Philadelphia, PA 19107 USA

## Abstract

The function of most long noncoding RNAs (lncRNAs) is unknown. However, recent studies reveal important roles of lncRNAs in regulating cancer-related pathways. Human antisense lncRNA-*NKX2-1-AS1* partially overlaps the *NKX2-1*/TTF1 gene within chromosomal region 14q13.3. Amplification of this region and/or differential expression of genes therein are associated with cancer progression. Herein we show higher levels of *NKX2-AS1* and *NKX2-1* in lung adenocarcinomas relative to non-tumor controls but no correlation between *NKX2-1-AS1* and *NKX2-1* levels across specimens, or with amplification of the 14q13.3 region, suggesting that *NKX2-1-AS1* and *NKX2-1* are independently regulated. Loss-and-gain of function experiments showed that *NKX2-1-AS1* does not regulate *NKX2-1* expression, or nearby genes, but controls genes in trans. Genes up-regulated by *NKX2-1-AS1*-knockdown belong to cell adhesion and PD-L1/PD-1 checkpoint pathways. *NKX2-1-AS1* negatively regulates endogenous *CD274*/PD-L1, a known target of *NKX2-1*, and the transcriptional activity of -1kb-*CD274* promoter-reporter construct. Furthermore, *NKX2-1-AS1* interferes with *NKX2-1* protein binding to the *CD274*-promoter, likely by *NKX2-1* protein-*NKX2-1-AS1* interactions. Finally, *NKX2-1-AS1* negatively regulates cell migration and wound healing, but not proliferation or apoptosis. These findings support potential roles of *NKX2-1-AS1* in limiting motility and immune system evasion of lung carcinoma cells, highlighting a novel mechanism that may influence tumorigenic capabilities of lung epithelial cells.

## Introduction

Genomic regions that are frequently amplified in human lung cancer often contain genes that control tumorigenesis. However, the contribution of each gene within these amplified genomic regions to the tumorigenesis process is often not fully defined^1^. For example, the 14q13.3 cytoband is a genomic region amplified in ~15% of lung cancers^2–4^ and mutations or deletions in this chromosomal region are frequently found in lung and thyroid cancer patients^5,6^. Within this region, the transcription factors *NKX2-1* (thyroid transcription factor-1; also known as *TTF1*), *NKX2-8* and *PAX9* are candidate genes that cooperate to control lung cancer cell growth, although other genes in this region are also likely to facilitate the tumorigenesis process^4,7^. Recently, by whole transcriptome sequencing, a few long non-coding RNAs have been identified that map to this region, including lnc-*NKX2-8* and lnc-*SLC25A21*^8,9^, *NANCI*^10^ and *NKX2-1-AS1*^11^; however, their contribution to lung tumorigenesis is not fully understood.

lncRNAs are transcripts of more than 200 nucleotides in length, but in contrast to protein-coding genes, they lack the structural features needed to be translated into proteins^12^. The functions of an increasing number of individual lncRNAs have been determined, and the dysregulation of lncRNAs has been linked to a number of diseases. However, for the majority of the annotated lncRNAs, the functional relevance is unknown^13^. The lack of knowledge about conserved sequences or structures among mammalian lncRNAs has made it difficult to infer the potential functions of these genes as has been done for many protein-coding genes. Efforts are in progress, though, to identify these domains in fungi^14^ and ultimately in vertebrates^15^. Therefore, studies about the functions of individual lncRNAs are warranted to determine their role as part of regulatory networks^16^. This is especially relevant for novel lncRNAs associated with human diseases. Recent studies have revealed the role of an increasing number of lncRNAs in regulating key cancer pathways at a transcriptional, post-transcriptional and epigenetic level^17–20^. Moreover, lncRNAs have been shown to be differentially expressed across various stages of tumor differentiation^19,21^. Evidence indicates that the lncRNA *NKX2-1-AS1* is specifically detected in carcinoma cell lines of lung origin^22^ and, like the adjacent protein-coding gene *NKX2-1*, is highly expressed in primary lung adenocarcinomas compared to squamous carcinomas^11,21,23^ and in some small cell carcinomas^24^, but its contribution to lung tumorigenesis is not well understood.

To evaluate the potential regulatory and biological functions of the *NKX2-1-AS1* transcript, we performed expression analyses, loss and gain of function experiments, and functional tests. We have uncovered in this study a role for the *NKX2-1-AS1* transcript in regulating genes that control cell adhesion and the migration of lung tumor cell lines. We also identified a role for *NKX2-1-AS1* in the regulation of *CD274*, the gene encoding the Programmed Death-Ligand 1 (PD-L1). These effects are independent of the transcription or translation of the adjacent gene encoding the transcription factor *NKX2-1*. These findings highlight a new mechanism by which *NKX2-1-AS1* might regulate tumorigenic properties of lung cells.

## Results

### *NKX2-1-AS1* and *NKX2-1* are co-expressed at variable levels in lung carcinoma cells, lung cell lines and normal tissues

**Figure 1:**
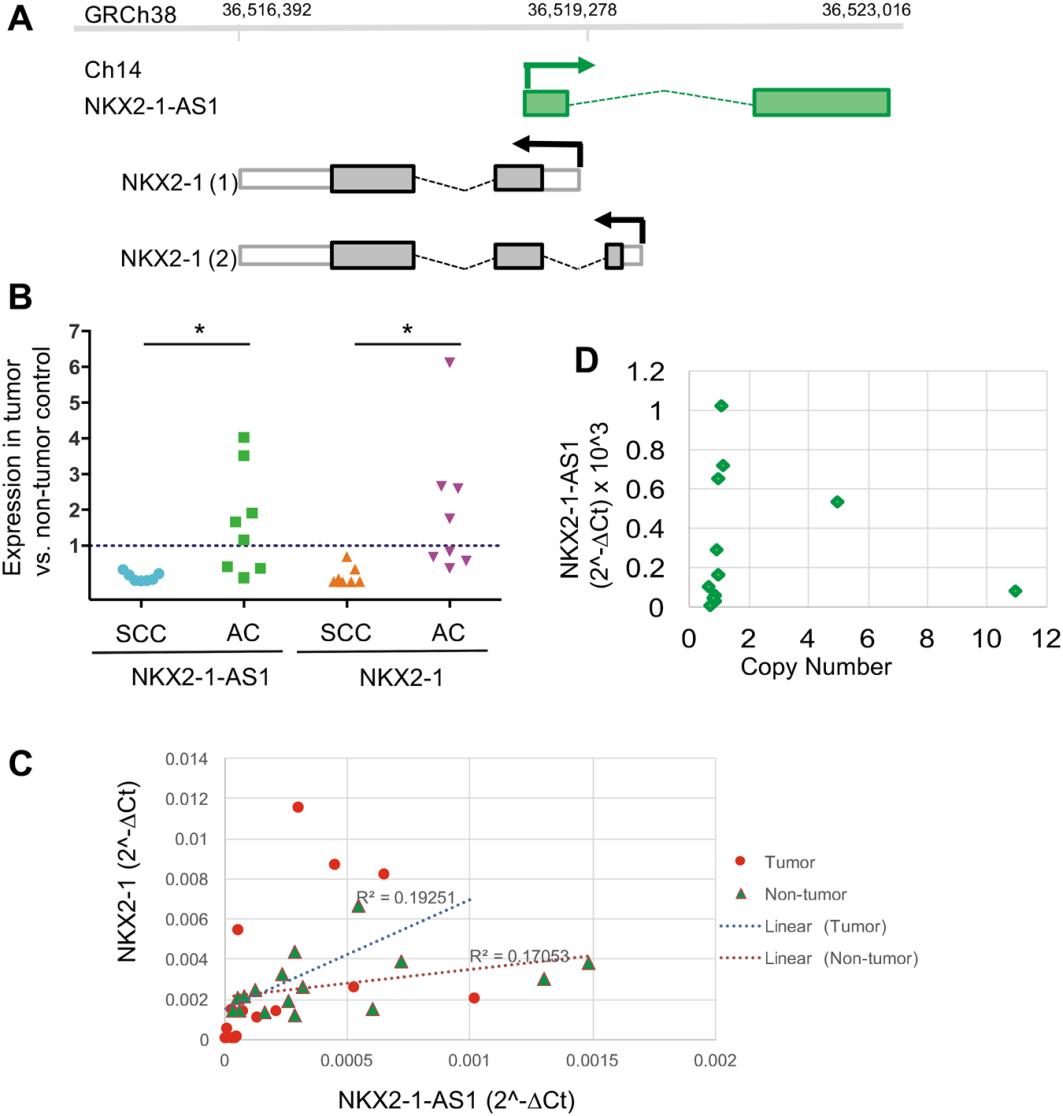
*NKX2-1-AS1* expression patterns in human non-small cell lung carcinoma (NSCLC). (**A**) Schematic representation of the relative chromosomal location of *NKX2-1-AS1* lncRNA and adjacent *NKX2-1* protein coding-gene in human chromosomal region 14q13.3. Arrows indicate direction of transcription. Boxes indicate exons, dotted lines indicate introns and colored boxes indicate coding regions. (**B**) Levels of expression of *NKX2-1-AS1* and *NKX2-1* in NSCLCs (SCC = squamous cell carcinoma; and AC = adenocarcinoma) relative to their corresponding non-tumor control determined by qPCR (n = 8; *p < 0.03). (**C**) Correlation plot of the expression of *NKX2-1-AS1* and *NKX2-1* determined by qPCR in the above tumors and their corresponding non-tumor specimens analyzed in this study. (**D**) Amplification status of the *NKX2-1-AS1* locus determined by qPCR of genomic DNA and expressed as copy number of the *NKX2-1-AS1* gene per genome plotted relative to *NKX2-1-AS1* expression level in each sample (n = 12).

Previous RNA-sequencing analyses of non-small cell lung carcinomas (NSCLCs) have shown that *NKX2-1-AS1* is highly expressed in primary lung adenocarcinomas compared to squamous carcinomas^11,21,23^ and in some small cell carcinomas^24^, a pattern of expression that is similar to that of the adjacent protein-coding gene *NKX2-1*. To validate whether the *NKX2-1-AS1* transcript annotated in public databases (ENST00000521292.2, hg38 chr14:36,519,278–36,523,016, Fig. 1A) is detected in lung tumors, we measured the expression of *NKX2-1-AS1* and *NKX2-1* using qPCR in a select number of lung adenocarcinomas (AC, n = 8) and squamous cell carcinomas (SCC, n = 8) and their matched non-tumor controls, described in Table 1. This analysis showed that, compared to non-tumor matching controls, *NKX2-1-AS1* and *NKX2-1* expression was increased in adenocarcinoma, whereas levels of both *NKX2-1-AS1* and *NKX2-1* were decreased in squamous cell carcinoma [AC vs SCC: *NKX2-1-AS1 p* = 0.030; *NKX2-1 p* = 0.029] (Fig. 1B), in concordance with previous RNA-sequencing findings that *NKX2-1-AS1* expression is higher in lung adenocarcinomas than in squamous cell carcinomas^11,21^. However, we noticed that the relative expression of *NKX2-1* and *NKX2-1-AS1* differed considerably across tumors. Furthermore, there was no significant correlation between the expression of these two genes across the specimens evaluated in this study (Fig. 1C), or across the tumors in a publicly available NSCLC expression GEO dataset (GDS3627)^25^ (Supplementary Fig. S1). Variable *NKX2-1-AS1* expression was observed in the presence of relatively constant *NKX2-1* expression, and *vice versa*. These results suggest that in certain conditions co-transcription of these genes is either biased towards one direction and/or that transcripts for these genes are degraded at different rates. Alternatively, these findings might suggest that *NKX2-1-AS1* and *NKX2-1* are independently regulated.

**Table 1:** Classification of the lung tumor samples used in the current studies*.

Tumor type	Differentiation stage			Age	Sex
Adenocarcinoma	AJCC G2: Moderately differentiated	pT1pN0pMX	IA	61	Female
	AJCC G2: Moderately differentiated	pT1pN0pMX	IA	77	Female
	AJCC G2: Moderately differentiated	pT2pN0pMX	IB	86	Female
	AJCC G2: Moderately differentiated	pT1pN1pMX	IIA	61	Male
	AJCC G2: Moderately differentiated	pT1pN1pMX	IIA	61	Male
	AJCC G2: Moderately differentiated	pT1pN1pMX	IIA	57	Female
	AJCC G3: Poorly differentiated	pT2pN2pMX	IIIA	72	Male
	AJCC G3: Poorly differentiated	pT4pN1pMX	IIIB	70	Male
	AJCC G2: Moderately differentiated	pT4pN1pMX	IIIB	60	Female
Squamous Cell Carcinoma	AJCC G3: Poorly differentiated	pT1pN0pMX	IA	62	Female
	AJCC G2: Moderately differentiated	pT1pN0pMX	IA	62	Male
	AJCC G2: Moderately differentiated	pT2pN0pMX	IB	68	Male
	AJCC G3: Poorly differentiated	pT2pNXpMX	IB	77	Male
	AJCC G2: Moderately differentiated	pT2pN1pMX	IIB	78	Male
	AJCC G2: Moderately differentiated	pT2pN1pMX	IIB	54	Male
	AJCC G2: Moderately differentiated	pT3pN1pMX	IIIA	76	Male
	AJCC G3: Poorly differentiated	pT2pN2pMX	IIIA	73	Male

*Total RNA and DNA samples from lung tumors and their matching non-tumor control were obtained from OriGene.

*NKX2-1-AS1* is located in the 14q13.3 chromosomal region, which is amplified in ∼15% of lung cancers^2,4^. To evaluate whether differences in the expression of *NKX2-1-AS1* in tumor samples was due to amplification of this locus, we analyzed the copy number of *NKX2-1-AS1* by genomic qPCR. We normalized the values to *LINE1*, a repetitive sequence with relatively conserved copy number in human normal and neoplastic cells^26,27^. Normal human iPSC DNA was used to generate the calibration curves. We found that all but two samples tested contained one copy of *NKX2-1-AS1* per genome (Fig. 1D). Moreover, the two samples with a higher copy number (5 or 11 copies) have lower *NKX2-1-AS1* expression than many samples with only one copy. Despite the variable expression levels of *NKX2-1-AS1* in the specimens studied, including some with very high levels of *NKX2-1-AS1*, a link between expression level and copy number was not observed.

**Figure 2:**
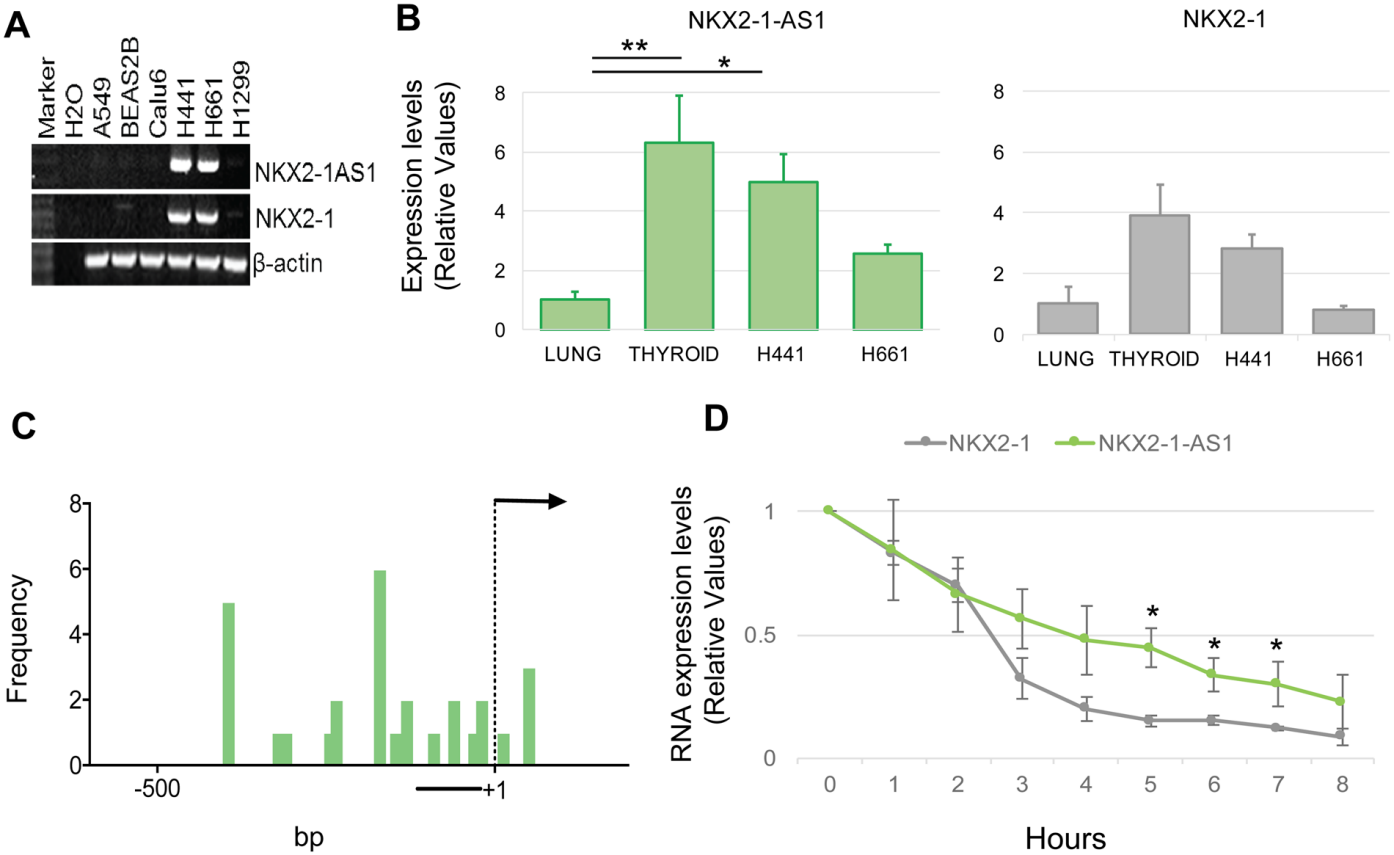
*NKX2-1-AS1* follows tissue-specific patterns of expression similar to *NKX2-1* in human cells. (**A**) Expression of *NKX2-1-AS1* and *NKX2-1* determined by RT-PCR in lung cell lines. The *NKX2-1-AS1* PCR fragments were sequenced to confirm the identity of the sequence. (**B**) Relative expression patterns of *NKX2-1-AS1* and *NKX2-1* in tissues and cell lines, including normal human adult lung and thyroid and H441 and H661 cell lines, as determined by qPCR (n = 3; *p < 0.05; **p < 0.01). (**C**) 5′-RACE analysis of *NKX2-1-AS1* in human thyroid identified multiple transcription initiation sites within the 500 bp 5′ of the transcription initiation site reported in Ensembl. Thyroid total RNA was used as it has higher levels of *NKX2-1-AS1* expression than the lung. The sensitivity of the method did not allow us to analyze lung RNA. Thirty clones, generated in three independent experiments, were sequenced. The black bar indicates the region recently identified in a genome wide analysis of accurate lncRNA transcription initiation sites^13^. (**D**) Time course analysis of *NKX2-1-AS1* and *NKX2-1* transcript stability in H441 cells by qPCR after inhibition of transcription by actinomycin D treatment (n = 3; *p < 0.05).

To determine whether *NKX2-1-AS1* is also expressed in cell lines known to express *NKX2-1*, we measured the expression of *NKX2-1-AS1* and *NKX2-1* by RT-PCR (Fig. 2A) in human lung carcinoma cell lines. Some lines, such as H441 and H661, express both *NKX2-1* and *NKX2-1-AS1*, but others, including H1299, BEAS-2B, A549 and Calu-6, do not express detectable levels of either transcript. We also quantified the relative level of expression of *NKX2-1-AS1* and *NKX2-1* in H441 and H661 cells relative to normal lung and thyroid (Fig. 2B). H441 cells express the highest levels of both genes among the cell lines tested; therefore, most studies were performed in this cell line, with confirmatory studies performed in H661 cells. Both genes are expressed at higher levels in the thyroid than in the lung (Fig. 2B).

These data indicate that the overall pattern of expression of *NKX2-1-AS1* mature transcript is similar to that of *NKX2-1* in normal and tumor tissues and cell lines, but that the relative expression levels of these genes vary among specimens, and that increased levels of expression of *NKX2-1-AS1* in certain lung tumors is not explained by locus amplification.

### *NKX2-1-AS1* transcripts can be initiated at multiple transcription start sites

Most lncRNA sequences are derived from RNA-sequencing assemblies, and in general, their 5′ ends are not accurate^13^. By 5′-RACE analyses of the *NKX2-1-AS1* transcripts in normal human thyroid, which has higher level of expression of *NKX2-1-AS1* compared to lung, we identified multiple transcription initiation sites for the *NKX2-1-AS1* transcript in a 500 bp region surrounding the TSS that has been reported in Ensembl (ENST00000521292.2, hg38 chr14:36,519,278) (Fig. 2C). These results are supported by a recent *in silico* study of the potential TSS of lncRNAs^13^, which found that most of the TSS for *NKX2-1-AS1* are located around 100 bp upstream of the TSS reported in Ensembl. These findings suggest the presence of longer *NKX2-1-AS1* transcripts that have not been identified in previous RNA-sequencing analyses.

### *NKX2-1-AS1* transcript is more stable than *NKX2-1* transcript

Since divergent transcription is a common phenomenon that can result in shorter transcripts that are rapidly degraded, or longer transcripts that might perform unique biological functions^28,29^, we measured the relative stability of the non-coding *NKX2-1-AS1* transcript compared to that of the protein-coding *NKX2-1* transcript. For these analyses, we inhibited transcription using actinomycin D and collected cells for RNA analysis by qPCR at different time points from 0 to 8 h. These analyses showed that despite the relatively low expression of *NKX2-1-AS1*, the transcript is more stable; 50% of the initial transcripts could be detected at more than 4 hours, whereas *NKX2-1* levels were reduced by 50% within approximately 2 hours (Fig. 2D).

### *NKX2-1-AS1* does not regulate expression of *NKX2-1* or other genes in the 14q13.3 chromosomal region

**Figure 3:**
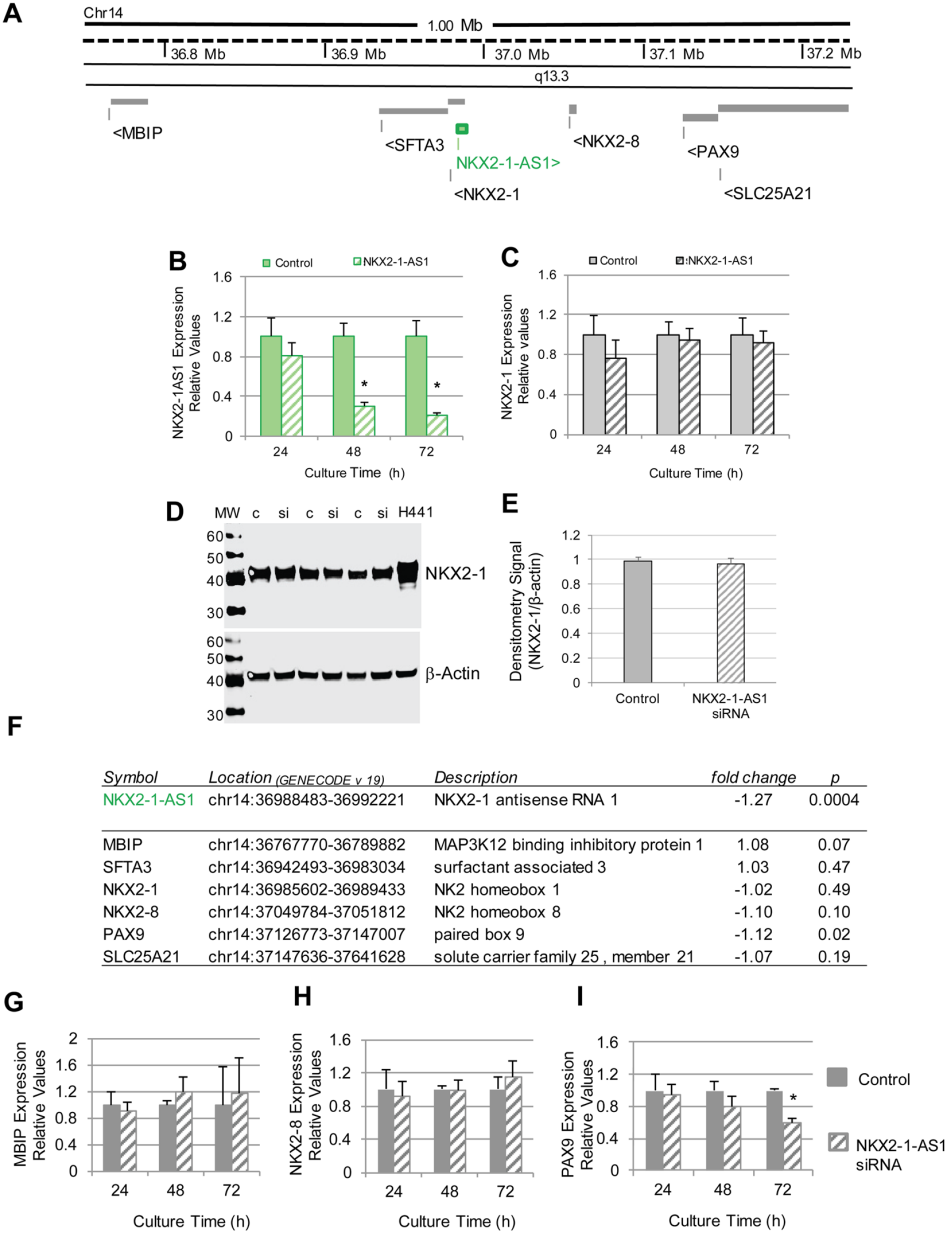
*NKX2-1-AS1* does not regulate expression of genes in the 14q13.3 chromosomal region. (**A**) Scheme of human chr14 within the 14q13.3 cytoband region indicating selected genes neighboring *NKX2-1-AS1*. (**B**) qPCR analyses of *NKX2-1-AS1* in H441 cells treated with a pool of three siRNAs targeting *NKX2-1-AS1* exon 2 show significant down-regulation of *NKX2-1-AS1* at 48 h and 72 h post transfection (n = 6; **p = 0.01 and ***p = 0.005 respectively). (**C**) qPCR analyses of the neighboring protein coding gene *NKX2-1* in *NKX2-1-AS1* knocked-down cells show no changes in *NKX2-1* RNA levels (n = 6). (**D**) Representative western blots of *NKX2-1* protein in non-silencing control [c] and *NKX2-1-AS1* siRNA [si] treated H441 cells normalized to β-actin. (**E**) Densitometry of the western blot signals normalized to β-actin indicates that *NKX2-1* protein levels also remained unchanged; n = 3. (**F**) Expression levels of *NKX2-1-AS1* in the knockdown cells at 48 h and of other genes in the 14q13.3 chromosomal region as determined by microarray analysis; n = 6. (**G**) qPCR analysis in the *NKX2-1-AS1* knockdown cells at 24, 48 and 72 h of *MBIP*; (H) *NKX2-8*; and (I) *PAX9*. n = 6, *p = 0.002.

LncRNAs can regulate genes within nearby or distant genomic regions. To evaluate whether the *NKX2-1-AS1* transcript regulates the expression of adjacent genes within the 14q13.3 genomic region (Fig. 3A), we performed loss of function experiments using a mix of three siRNAs targeting *NKX2-1-AS1* and a non-silencing control in H441 and H661 cells. Using qPCR, we showed that reduction of *NKX2-1-AS1* expression by ~70% at 48 h or by ~90% at 72 h in H441 cells using the mix of three siRNAs (Fig. 3B) did not affect expression of *NKX2-1* at the mRNA (Fig. 3C) or protein (Fig. 3D,E) level. Individual siRNAs were also tested and no significant down-regulation of *NKX2-1* was observed in H441 cells (Supplementary Fig. S2). Similarly, no reduction in *NKX2-1* mRNA levels was observed in H661 cells after knockdown of *NKX2-1-AS1* (Supplementary Fig. S3).

We also measured gene expression following *NKX2-1-AS1* knockdown in H441 cells using microarrays, which showed that the expression of most genes in the 14q13.3 cytoband (Fig. 3A), including *MBIP, SFTA3 (NANCI), NKX2-8*, and *SLC25A21*, was unchanged at 48 h, with the exception of a small but significant effect on *PAX9* (p = 0.02, FDR q = 0.17) (Fig. 3F). We validated these findings by qPCR at 24, 48 and 72 h after knockdown of *NKX2-1-AS1* (Fig. 3G–I). Down-regulation of PAX9 was observed, but only at 72 h; expression of the other genes in the cytoband was not changed. Overall, these data support a limited role of the *NKX2-1-AS1* transcript in regulating genes in cis.

### *NKX2-1-AS1* does not control alternative splicing of *NKX2-1* RNA or translation of *NKX2-1* protein

Analyses of the role of divergent lncRNAs with 5′ regions that overlap a protein-coding mRNA have demonstrated that some of them can control alternative splicing and/or protein translation^30^. As shown in Fig. 3D,E, no changes in *NKX2-1* protein levels were observed in cells treated with *NKX2-1-AS1* siRNAs for 48 h, despite a 70% reduction in *NKX2-1-AS1* levels. Furthermore, no changes in the ratio of the two major *NKX2-1* variants (variant 1 and variant 2) were detected by RT-qPCR (data not shown). These data suggest that the partial overlapping of the 5′ ends of *NKX2-1-AS1* and *NKX2-1* does not control the selection of different transcription initiation sites, splicing of *NKX2-1* RNA, or translation of *NKX2-1* protein, as has been shown for other divergent overlapping mRNA-lncRNA pairs^30^.

### Overexpression of *NKX2-1* does not change the expression levels of *NKX2-1-AS1*

To evaluate whether *NKX2-1* had any effect on *NKX2-1-AS1* expression, we performed *NKX2-1* gain of function experiments in H441 cells. We found that overexpression of *NKX2-1* by more than 300-fold did not result in alterations in the expression of endogenous *NKX2-1-AS1*, but significantly increased expression of the positive control target gene *SFTPC* by 2.7-fold. These data suggest that *NKX2-1* and *NKX2-1-AS1* do not have a direct reciprocal transcriptional regulatory relationship (Supplementary Fig. S4).

### *NKX2-1-AS1* negatively regulates PD-1/PD-L1 signaling and adherens junction pathways

**Figure 4:**
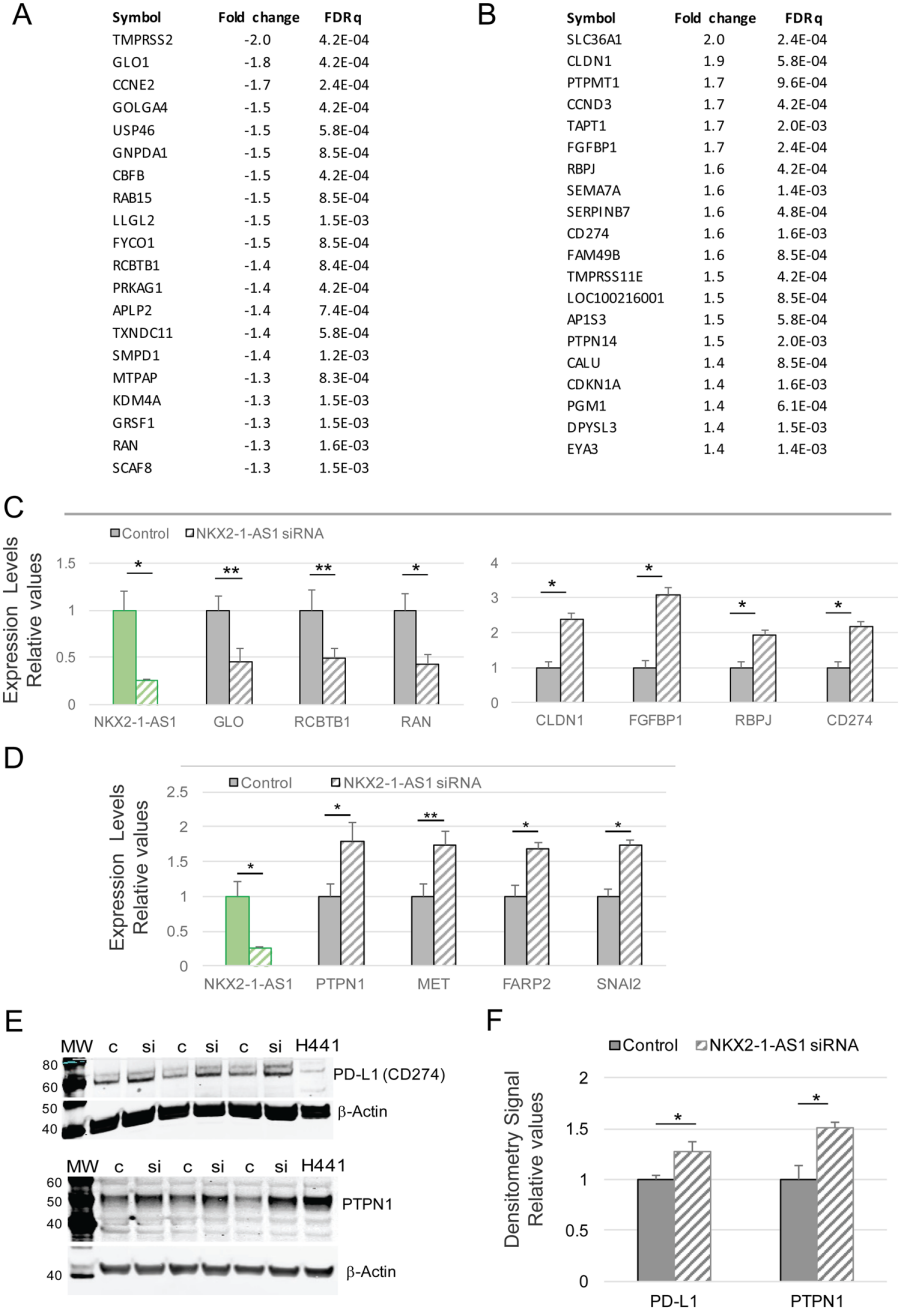
*NKX2-1-AS1* knockdown alters gene expression patterns in H441 cells. (**A**) List of the top 20 genes down- and (B) up-regulated by *NKX2-1-AS1* knockdown determined by microarray analysis, 48 h after treatment (n = 6). (**C**) qPCR validation of down-regulated and up-regulated genes in *NKX2-1-AS1* knockdown cells at 48 h after treatment (n = 3, *p < 0.05, **p < 0.01). (**D**) Real time PCR validation of adherens junction related genes up-regulated by *NKX2-1-AS1* knockdown (n = 3; *p < 0.05; **p < 0.01). (**E**) Representative western blots of PD-L1 and PTPN1 protein in non-silencing control [c] and *NKX2-1-AS1* siRNAs [si] treated H441 cells normalized to β-actin. (**F**) Densitometry of the western blot signals normalized to β-actin indicates that PD-L1 (n = 9; **p = 0.002) and PTPN1 (n = 3; *p = 0.05) protein levels are increased by *NKX2-1-AS1* knockdown.

The microarray experiment showed considerable changes in gene expression after 48 h of *NKX2-1-AS1* knockdown in H441 cells (140 genes at FDR q < 0.01. The top twenty genes that were down-regulated or up-regulated by *NKX2-1-AS1* knockdown are listed in Fig. 4A,B. Results for selected genes within these lists were validated by RT-qPCR (Fig. 4C). GSEA was then used to identify biological terms, pathways and processes that were coordinately up- or down-regulated after *NKX2-1-AS1* knockdown (Supplementary Materials). Two of the gene sets with the most significant coordinate up-regulation following *NKX2-1-AS1* silencing are the Reactome PD-1 signaling and KEGG adherens junction pathways (p < 0.001, FDR q < 0.25). Validation of selected genes in the adherens junction pathway (Supplementary Material) are shown in Fig. 4D.

The PD-1 signaling pathway includes the gene *CD274*, which encodes PD-L1. The up-regulation of *CD274* mRNA and PD-L1 protein following *NKX2-1-AS1* knockdown was validated in H441 cells by qPCR (Fig. 4C) and western blot (Fig. 4E,F) respectively and in H661 cells by RT-qPCR (Supplementary Fig. S5A). An inverse trend between the regulation of *NKX2-1-AS1* and PD-L1 in tumors relative to non-tumor controls is observed in the specimens tested in this work as well as in a publicly available GEO dataset (GDS3627) (Supplementary Fig. S6). The up-regulation of genes involved in adherens junction formation, including *PTPN1* (protein tyrosine phosphatase, non-receptor type 1) that promotes migration and invasion in breast cancer cells^31^ and in epidermal dendritic cells^32^*, FARP2* (FERM, ARH/RhoGEF and pleckstrin domain protein 2), *SNAI2* (snail family transcriptional repressor 2), and *MET* (MET proto-oncogene, receptor tyrosine kinase), was validated by qPCR in H441 cells (Fig. 4D), and the up-regulation of *MET* was also validated by qPCR in H661 cells (Supplementary Fig. S5B), suggesting a potential role for *NKX2-1-AS1* in the regulation of cell-cell interaction and motility processes. Furthermore, *CLDN1*, a gene encoding a tight junction component, was also up-regulated by *NKX2-1-AS1* knockdown in both cell lines (Fig. 4C and Supplementary Fig. S5A).

### *NKX2-1-AS1* impairs binding of *NKX2-1* protein to the *CD274* gene promoter

**Figure 5:**
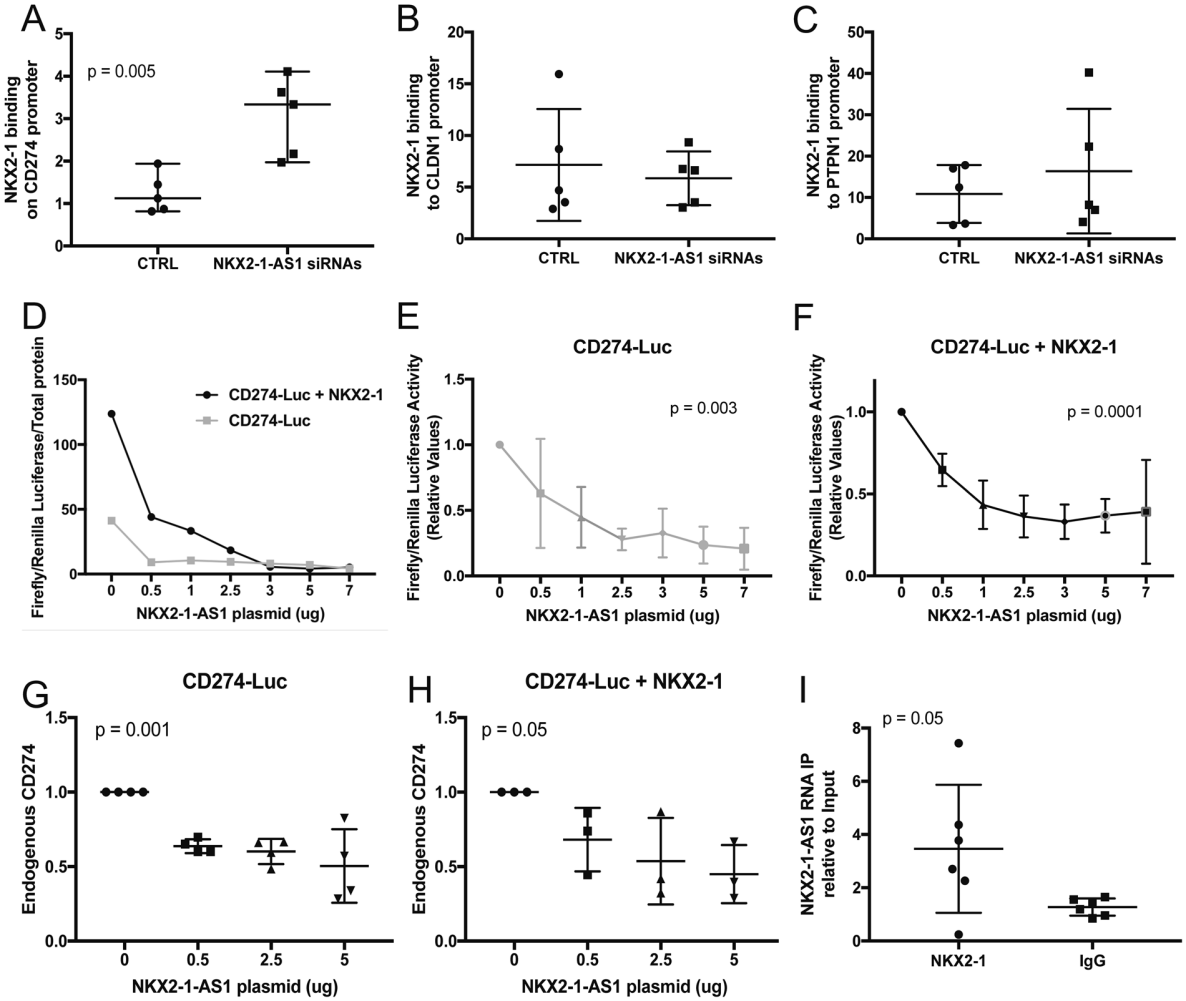
*NKX2-1-AS1* overexpression reduces *CD274* expression levels in A549 cells in part by impairing *NKX2-1* protein binding to the *CD274* promoter. ChIP-qPCR analysis of NKX2-1 protein binding to (**A**) *CD274* promoter (n = 4; p = 0.005), (**B**) *CLDN1* promoter (n = 5), and (**C**) *PTPN1* promoter (n = 5) in H441 cells transfected with *NKX2-1-AS1* siRNAs or non-silencing control. (**D**) *NKX2-1* co-transfection with the −1kb*CD274*-Luc vector results in higher luciferase activity (3-fold in the absence of *NKX2-1-AS1*, 0ug). *NKX2-1-AS1* overexpression reduces the activity of the −1kb*CD274* promoter in a dose-dependent manner both in the absence (**E**) (n = 3-4; ANOVA *p* = 0.003) or presence (**F**) (n = 3-4; ANOVA *p* = 0.0001) of *NKX2-1* overexpression. *NKX2-1-AS1* overexpression reduces the expression of the endogenous *CD274* gene in a dose-dependent manner both in the absence (**G**) (n = 3-4; ANOVA *p* = 0.001) or presence (**H**) (n = 3-4; ANOVA *p* = 0.05) of *NKX2-1* overexpression. (I) RIP-qPCR analysis of *NKX2-1-AS1* pull down by *NKX2-1* antibody compared to IgG control (n = 6; *p* = 0.05).

To identify potential mechanisms of negative regulation of *CD274* by *NKX2-1-AS1*, we used ChIP-qPCR to evaluate the effect of *NKX2-1-AS1*-knockdown on the binding of *NKX2-1*, which is known to directly activate the transcription of *CD274*^33^. Reducing the expression of *NKX2-1-AS1* by siRNA resulted in increased binding of *NKX2-1* protein (Fig. 5A) (n = 5, *p* = 0.005) to the *CD274* promoter region. However, the binding of *NKX2-1* protein to the proximal promoters of *PTPN1* and *CLDN1*, which were also negatively regulated by *NKX2-1-AS1*, was unchanged (Fig. 5B,C).

### *NKX2-1-AS1* inhibits transcription from the *CD274* proximal promoter and reduces *NKX2-1*-mediated activation of *CD274* transcription

To confirm that *NKX2-1* increased *CD274* expression by direct activation of the *CD274* proximal promoter, we performed co-transfection experiments in A549 cells with plasmids containing *NKX2-1* or *NKX2-1-AS1* overexpression constructs and a luciferase reporter driven by 1.1 kb of the *CD274* promoter. *NKX2-1* overexpression increased the activity of the *CD274* reporter by 3-fold (Fig. 5D, 0 ug). Conversely, *NKX2-1-AS1* overexpression reduced the activity of the *CD274* reporter in a dose-dependent manner, both in the absence (ANOVA *p* = 0.003) and presence (ANOVA *p* = 0.0001) of *NKX2-1* overexpression (Fig. 5E,F, respectively). Furthermore, *NKX2-1-AS1* overexpression reduced the expression of endogenous *CD274* in a dose-dependent manner, both in the absence (ANOVA *p* = 0.001) and presence (ANOVA *p* = 0.05) of *NKX2-1* overexpression (Fig. 5G,H, respectively). *NKX2-1-AS1* overexpression also reduced the expression of endogenous *CLDN1* (ANOVA *p* = 0.0002) in a dose-dependent manner, but did not affect the expression of *PTPN1*, *RBPJ* or *FARP2* (Supplementary Fig. S7).

### *NKX2-1-AS1* interacts with *NKX2-1* protein

One of the mechanisms by which lncRNAs inhibit gene expression is by acting as decoys and preventing binding of transcription factors to DNA^34^. To evaluate whether *NKX2-1-AS1* controls the binding of *NKX2-1* to the *CD274* promoter, we used RNA immunoprecipitation followed by qPCR (RIP-qPCR) to investigate the potential interaction of the *NKX2-1-AS1* lncRNA and *NKX2-1* protein. In H441 cells, endogenous *NKX2-1-AS1* co-immunoprecipitated with *NKX2-1* protein (*p = *0.05 versus IgG control) (Fig. 5I), suggesting that *NKX2-1-AS1*-*NKX2-1* interactions may inhibit *NKX2-1* binding to *CD274* promoter.

### *NKX2-1-AS1* does not change the histone methylation landscape of the *CD274* promoter

Another mechanism by which lncRNA mediates the inhibition of gene expression is through the recruitment of chromatin-modifying proteins to alter epigenetic marks on gene regulatory regions^34^. To evaluate this possibility, we used ChIP in H441 cells to measure H3K4me3 (active chromatin) and H3K27me3 (silenced chromatin) marks and binding of the histone 3 lysine 27 methyltransferase EZH2 to the promoters of *CD274, CLDN1*, and *PTNP1* (which were up-regulated by the knockdown of *NKX2-1-AS1*) and the *NKX2-1* promoter. We found that only H3K4me3 was enriched above IgG background levels at these promoters, and no significant changes in the enrichment of any of these marks was observed in *NKX2-1-AS1*-knocked down cells (Supplementary Fig. S8). These data suggest that *NKX2-1-AS1* inhibition of *CD274* is not linked to epigenetic gene regulation by histone methylation mechanisms.

### *NKX2-1-AS1* inhibits cell migration but has no significant effect on proliferation, invasion or apoptosis

**Figure 6:**
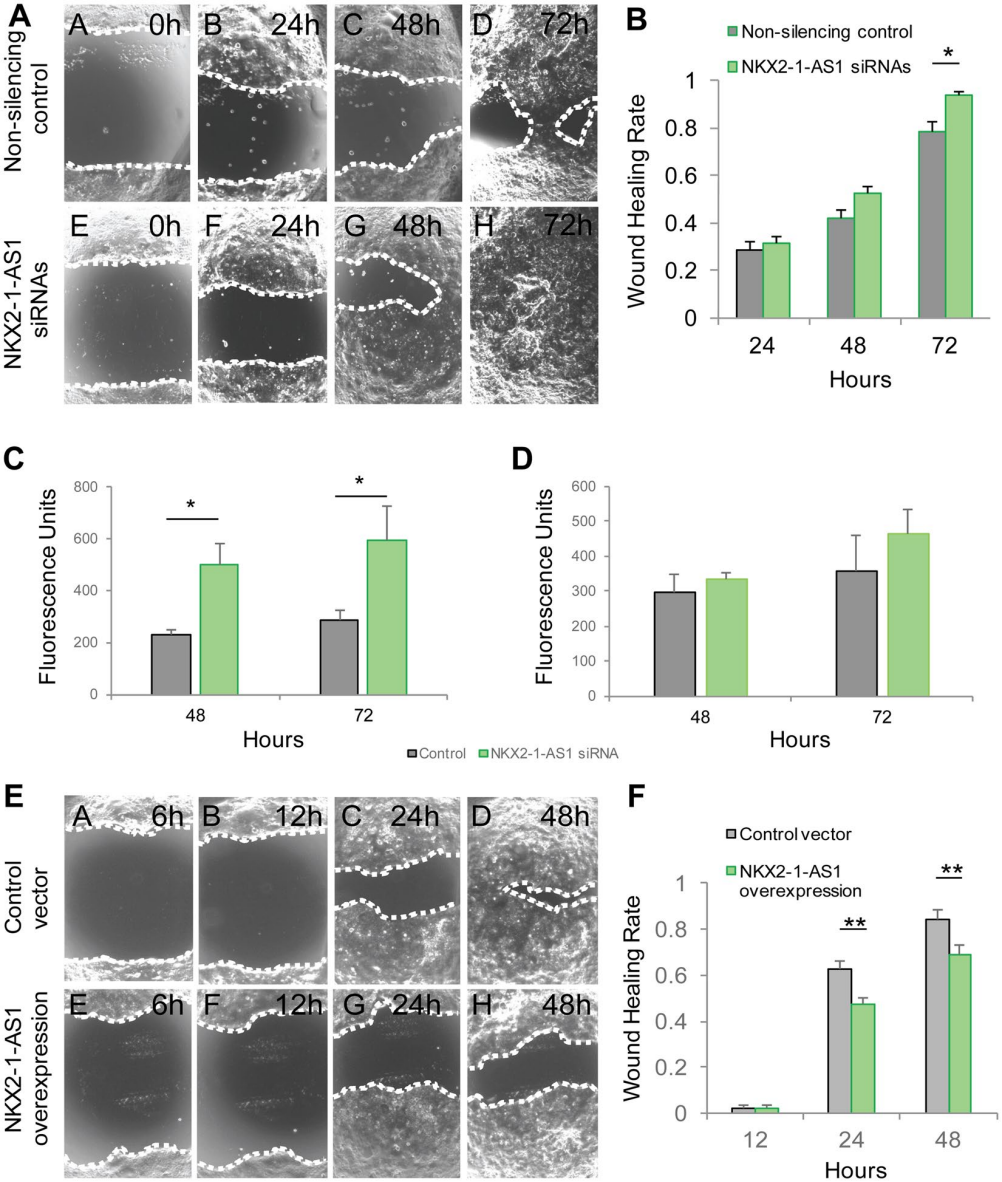
*NKX2-1-AS1* inhibits cell motility in H441 cells. (**A**) Representative wound healing analysis of H441 cells treated with *NKX2-1-AS1* siRNAs or non-silencing siRNA control. Cells were treated with the siRNAs for 24 h before the scratch was performed (0 h). Three images per scratch were taken at 0, 24, 48 and 72 h in 3 independent experiments. (**B**) Average wound area closed determined in the above images (*p < 0.002). (**C**) Migration of *NKX2-1-AS1* knockdown H441 cells was compared to non-silencing control in transwell experiments (n = 3; *p < 0.05). (**D**) Invasion of H441 cells treated with *NKX2-1-AS1* siRNA compared to non-silencing control in collagen-covered transwells (n = 3). (**E**) Representative wound healing analysis of H441 cells transfected with CMV-*NKX2-1-AS1* or with empty vector control. After treatment for 24 h the scratch was performed (0 h). Three images per scratch were taken at 0, 24, and 48 h in three independent experiments. (**F**) Average wound area closed (**p < 0.00001).

Because of the changes observed in genes involved in cell-cell interaction, we evaluated whether changes in *NKX2-1-AS1* expression levels have any effect on the motility of lung tumor cells. Using the mix of three siRNAs described above, we reduced *NKX2-1-AS1* expression by 70-90%. Cell monolayer scratch (wound healing) assays performed on these cells showed that decreasing *NKX2-1-AS1* expression produced a statistically significant (*p* < 0.002) increase in wound healing activity of these cells, underscoring a role in cell motility (Fig. 6A,B). Furthermore, migration and invasion analysis using polypropylene or extracellular matrix covered trans-wells showed that reduction of *NKX2-1-AS1* significantly increased migration of H441 cells (Fig. 6C) but had no effect on cell invasion (Fig. 6D). Conversely, overexpression of *NKX2-1-AS1 cDNA* by more than 100-fold in H441 cells by transient transfection did not change the level of expression of *NKX2-1* mRNA (Supplementary Fig. S4) but resulted in a moderate but significant reduction in wound healing activity (*p* < 0.00001) (Fig. 6E,F), supporting a role for *NKX2-1-AS1* transcript in the inhibition of lung tumor cell migration by mechanisms independent of regulation of *NKX2-1* expression.

**Figure 7:**
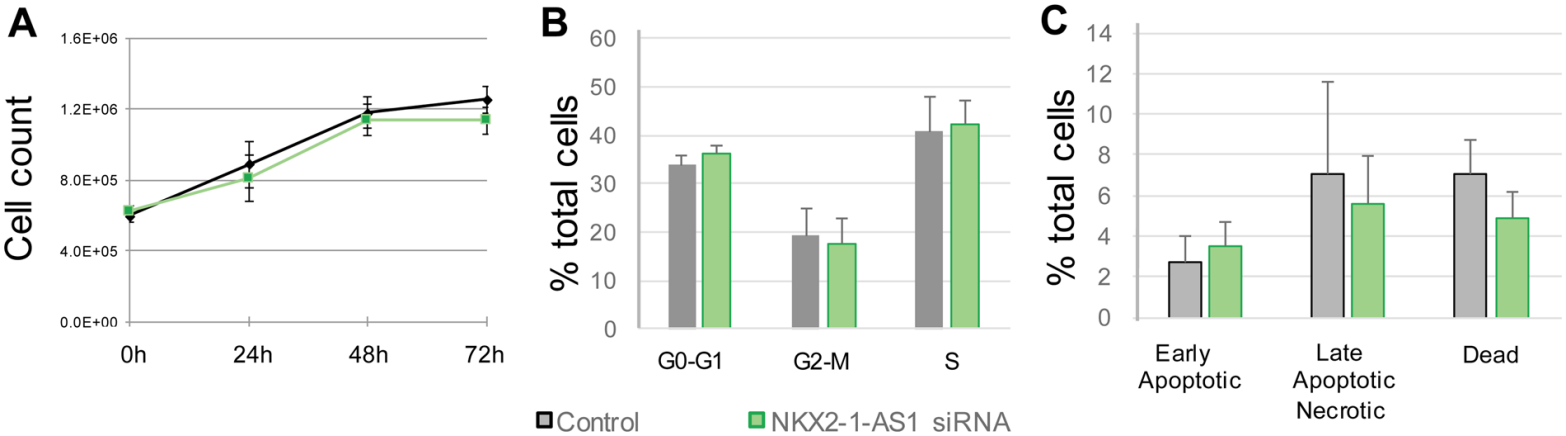
*NKX2-1-AS1* knockdown does not affect proliferation or apoptosis of H441 cells. (**A**) Cell growth was determined by counting cells at 24, 48 and 72 h after treatment with a pool of 3 siRNAs targeting *NKX2-1-AS1* (n = 3). (**B**) Analysis of cell cycle stage by measuring DNA cell content by flow cytometry in *NKX2-1-AS1* knockdown cells compared to non-silencing control at 48 h after treatment (n = 3). No significant change in cell number in each cell cycle stage was observed in *NKX2-1-AS1* knockdown cells compared to non-silencing control. (**C**) No significant change in apoptosis was observed in *NKX2-1-AS1* knockdown cells compared to non-silencing control as measured by annexin-V binding.

To further evaluate potential biological functions of the *NKX2-1-AS1* transcript, we tested whether *NKX2-1-AS1* controls other classic tumorigenic properties of lung epithelial cells, including cell growth, cell cycle progression and apoptosis. Reduction of *NKX2-1-AS1* by 70-90% using the mix of three siRNAs as described above resulted in minimal, and not statistically significant, cell growth reduction at 48 or 72 h (Fig. 7A). Cell cycle analysis of these samples by flow cytometry did not show changes in the number of cells in each stage of the cell cycle (Fig. 7B) or an increase in apoptosis as measured by annexin-V binding (Fig. 7C).

## Discussion

Because the 14q13.3 chromosomal region is believed to contribute to the pathobiology of lung tumorigenesis, we sought to elucidate the potential contribution of newly identified genes residing within this region to lung tumorigenesis. Recent findings point to a potential role for *NKX2-1-AS1*, a long non-coding RNA (lncRNA) mapped to this region, as a regulator of lung tumor cell properties: its selective expression in cell lines of lung and thyroid origin^22^; its increased expression in lung adenocarcinomas^11,21,23^; and its close proximity to the transcription factor *NKX2-1* (thyroid-transcription factor 1, *TTF1, NKX2.1*), which is already known to contribute to lung cancer progression^35^. In this study, we report our novel findings on the potential regulatory and biological functions of the *NKX2-1-AS1* transcript in lung cancer cells.

Despite the fact that the 14q13.3 chromosomal region, where *NKX2-1-AS1* and *NKX2-1* are located, is amplified in ~15% of adenocarcinomas^2,4^ and is the highest focally amplified region in lung adenocarcinomas^1^, we found no link between *NKX2-1-AS1* expression levels and the copy number of its locus. Although we cannot rule out a role for amplification in the expression of these genes in other lung tumor samples, our data suggest that an active mechanism of gene regulation is in place that controls *NKX2-1-AS1* expression in cells where the 14q13.3 region is not amplified. The relatively higher stability of the *NKX2-1-AS1* transcript observed in our studies might be one such factor contributing to the increased levels of this gene in lung adenocarcinomas.

Due to their relative position, the *NKX2-1* and *NKX2-1-AS1* genes are classified as bidirectional and divergent (transcribed from opposite strands in opposite directions)^29^. Moreover, the 5′ regions of the *NKX2-1* and the *NKX2-1-AS1* transcripts overlap, sharing > 300 bp of complementary sequence. The lncRNAs that are divergent to protein coding genes are thought to arise as a consequence of the transcription process in which the polymerase II works bidirectionally^36^. These antisense, noncoding transcripts are in many instances short RNA forms that are rapidly degraded. However, longer lncRNAs (>1-2 kb), which are frequently detected as spliced forms, do not degrade as readily and might have independent functions^37,38^. Although dissecting these two processes is difficult, demonstrating the relevance of the lncRNA transcript (e.g., *NKX2-1-AS1*) to gene regulation and cellular behavior could help recognize multiple functions that are attributed to a particular genomic region (e.g., 14q13.3 in lung tumorigenesis).

In this study, we found evidence for a role for the *NKX2-1-AS1* transcript in the regulation of lung gene expression. We were able to detect *NKX2-1-AS1* transcript by various methods, including qPCR and 5′-RACE, and showed that this transcript is more stable than the *NKX2-1* transcript. Furthermore, *NKX2-1-AS1* transcripts are initiated at multiple start sites within a 500 bp window. We found that loss of *NKX2-1-AS1* expression does not affect expression of genes in *cis* (including *NKX2-1*) with the exception of *PAX9*, a gene ∼140 kb 3′ to *NKX2-1-AS1*, which is moderately regulated by reduction of *NKX2-1-AS1* levels. Instead, the *NKX2-1-AS1* transcript affects the expression of genes in *trans*; in particular, genes involved in cell adhesion and in PD-L1/PD-1 signaling pathways in different cell lines were upregulated following *NKX2-1-AS1* knockdown.

Of particular interest was the finding that *NKX2-1-AS1* inhibits the expression of *CD274* (PD-L1) mRNA and protein. PD-L1 is expressed in tumors of many different origins, including NSCLC, and binds to the programmed death-1 (PD-1) receptor expressed on T-cells, B-cells, dendritic cells and natural killer T-cells^39^. High PD-L1 expression enables cancers to evade the host immune system^40^, thereby indirectly making them more aggressive. In fact, NSCLC patients with high PD-L1 expression exhibit poor overall survival^41^ and anti-PD-L1 and/or anti-PD-1 are now being used to treat many forms of cancer^42,43^, although only approximately 20% of patients respond to this type of treatment.

It has been previously shown that *NKX2-1* positively regulates *CD274* expression^33^. Now we show that *NKX2-1-AS1* negatively regulates *CD274* transcription by interfering with the recruitment of *NKX2-1* protein to the *CD274* promoter. As we have shown that *NKX2-1-AS1* is immunoprecipitated with *NKX2-1* protein, it is possible that *NKX2-1-AS1* acts as a decoy, preventing the binding of *NKX2-1* protein to the *CD274* promoter. Further studies will be focused on analyzing these specific interactions.

We also observed that reducing the expression of *NKX2-1-AS1* increases migration of tumor cells with minimal effect on cell proliferation. This role for *NKX2-1*-AS1 appears to be mediated through its suppression of the transcription of several genes involved in cell adhesion, including *PTPN1*, which promotes migration and invasion in breast cancer cells^31^ and in epidermal dendritic cells^32^ and dephosphorylates β-catenin, adding to the adhesive properties of the cells by activation of cadherin^44^, and *CLDN1*, which encodes a tight junction component necessary for cell migration in skin wound healing and in human lung carcinoma cells^45,46^. This suggests that levels of *NKX2-1-AS1* can modulate cell motility, a tumorigenic property that controls the dissemination and spreading of primary tumors.

Interestingly, however, the knockdown of *NKX2-1-AS1* did not result in reduced cell growth, cell cycle arrest, or an increase in apoptosis. These results stand in contrast to those of a recent study by Balbin *et al*.^11^, which suggested a role for *NKX2-1-AS1* in cell growth, since the down-regulation of *NKX2-1-AS1* by siRNAs that were individually transfected in H441 cells produced changes in cell number up to 100 h in culture. In initial experiments, we had tested the effect of reducing *NKX2-1-AS1* by individual siRNAs, and also observed a significant reduction in cell growth at 72 h post transfection (data not shown). However, in order to reduce non-specific effects of the siRNAs, we combined three different siRNAs, each of which was able to reduce *NKX2-1-AS1* expression>50%, and the combination of which resulted in 90% reduction in *NKX2-1-AS1* at 72 h post transfection. Because the effect of this combination on cell number was not significant in our study, it suggests that an experiment using only one siRNA might produce off-target effects.

In this study, we have characterized molecular and functional features of the *NKX2-1-AS1* mature transcript. However, we cannot establish whether the process of *NKX2-1-AS1* transcription itself controls the transcription of *NKX2-1* or *vice versa*, or whether this event has regulatory or biological relevance. Answering these questions will require a different approach. These studies will be the focus of future studies. For example, the transcription of the lncRNA *HAND2-AS1* (upperhand, Uph), which is divergent to the heart developmental transcription factor *HAND2*, has been shown to be necessary for *HAND2* transcription by establishing a permissive chromatin environment; in that work, however, the function of the Uph mature transcript itself was not evaluated^47^.

Further understanding of the mechanisms regulating PD-L1 expression could lead to new approaches to treat aggressive malignancies^48^. Our data suggest that high levels of *NKX2-1-AS1* are important for maintaining low levels of PD-L1 expression, presumably to limit the capacity of tumor cells to evade the immune system defenses.

In summary, this study is the first to show the role of the *NKX2-1*-AS1 transcript in lung gene regulation in the context of human lung cancer. Increased expression of *NKX2-1*-*AS1* in lung carcinomas might be a protective mechanism by which cancer cells limit cell migration, metastatic spread and immune system evasion. These findings, therefore, could lead to the discovery of new mechanisms to target in future therapeutic interventions.

## Methods

### Cell lines

*NKX2-1-AS1* is specifically detected in carcinoma cells of lung origin^22^. We selected for our studies human lung carcinoma cell lines that are known to express both *NKX2-1* and *NKX2-1-AS1*: NCI-H441 (derived from a papillary adenocarcinoma of the lung) and NCI-H661 (derived from lung epithelial cells from large cell lung cancer metastasized to lymph nodes). We used as negative controls cells that express low or undetectable levels of *NKX2-1* nor *NKX2-1-AS1*: NCI-H1299 (derived from NSCLC cells metastasized to lymph nodes) and A549 cells (derived from a lung epithelial carcinoma). In addition, we tested the expression in Calu-6 (derived from an anaplastic carcinoma of lung origin) and BEAS-2B (a virally transformed lung/bronchus epithelial cell line) [American Type Culture Collection (ATCC)]. Cells were grown in the conditions recommended by ATCC. The 14q13.3 chromosomal region is known to be amplified in NCI-H661 and not amplified in NCI-H441^4^.

### RNA isolation and qPCR

RNA purification, reverse transcription and quantitative real-time PCR (qPCR) were performed as described previously using the OneStep Real-Time PCR System (Applied Bio-systems)^49^. Data was normalized to β-actin. qPCR assays are listed in Supplementary Table S1. The identity of *NKX2-1* and *NKX2-1-AS1* qPCR products was confirmed by DNA sequencing.

### Human lung samples

Total RNA and the corresponding genomic DNA from NSCLC and adjacent control tissue were purchased from OriGene Technologies, Inc. Sample diagnosis was verified at OriGene by an independent board-certified pathologist. Patient demographic and clinical information for each sample were provided by OriGene. Normal human lung and thyroid total RNAs were purchased from Clontech/Takara Bio USA, ThermoFisher and Zymogen.

### 5′ RACE analysis

Rapid Amplification of cDNA Ends (RACE) was used to identify the 5′ ends of the *NKX2-1-AS1* transcript in primary human samples. Human thyroid RNA was used as this tissue has higher levels of *NKX2-1-AS1* expression than the lung and was sufficient for the detection limits of this assay. Total RNA (1ug) was used for first strand cDNA synthesis using Smarter® RACE 5′ (Takara/Clontech Inc.) following the manufacturer’s instructions with modifications. In the rapid amplification step with the *NKX2-1-AS1* specific primer GSP1 (Supplementary Table S1) we increased the number of cycles to 30 to improve amplification of transcripts with low expression. To reduce the amplification of unwanted products during the sequential nested PCR with the specific *NKX2-1-AS1* nested primer NGSP1 (Supplementary Table S1), the annealing temperature was increased to 70 °C and the number of cycles to 30. PCR products were separated by gel electrophoresis and the fragments purified using a gel extraction kit (Qiagen). The fragments were cloned into the vector provided in the RACE kit. DNA from isolated clones was purified using the QIAprep kit (Qiagen) and sequenced using NGSP1 primer, M13F, and M13R primers from the vector.

### *NKX2-1* RNA isoform analysis

By qPCR we analyzed the level of expression of the major variants of *NKX2-1* mRNA. One TaqMan assay spanning exons 1 and 2 of *NKX2-1* variant 1 and exons 2 and 3 of variant 2 was used to determine the level of the main *NKX2-1* mRNA variants. A second TaqMan Assay spanning exons 1 and 2 of variant 2 was used to detect only *NKX2-1* variant 2 (Supplementary Table S1).

### RNA half-life analysis

To measure *NKX2-1-AS1* half-life, NCI-H441 cells were seeded into 6-well plates at a density of 2 × 10^5^ cells/well and cultured for 24 hours to reach 60% confluency. To inhibit transcriptional activity, 5 mg/ml of actinomycin D (Sigma) were added to the medium. Cells were harvested at 15 min, 30 min, 45 min, 1 hr, 2 hr, 3 hr, 4 hr, 5 hr, 6 hr, 8 hr, and 24hrs. Total RNA was purified using RNeasy kit (Qiagen) and treated with DNA-Free kit (Qiagen). Isolated total RNA (1ug) was reverse transcribed (RT) and analyzed by qPCR using the *NKX2-1-AS1* and *NKX2-1* TaqMan assays (Supplementary Table S1).

### RNAi gene knockdown

We performed siRNA transfections to down-regulate *NKX2-1-AS1* levels. We tested four different custom made Silencer® Select siRNAs designed to target the second exon of *NKX2-1-AS1* and the region of the first exon not overlapping with the *NKX2-1* sequence *NKX2-1-AS1* siRNA A, B, C and D (Supplementary Table S1). We selected the three siRNAs (A, B and D) that individually caused >50% down regulation of *NKX2-1-AS1* at 48 h post transfection. The mix of these three siRNAs resulted in a reduction of >90% of *NKX2-1-AS1* levels at 72 h in culture. We seeded 150,000 cells/well in 6-well plates. Using Lipofectamine RNAiMAX (Thermo Fisher Scientific), 10uM of each siRNA or a non-silencing negative control RNA were added in Opti-MEM to 70% confluent cells. Cells were harvested at 24, 48, and 72 h post transfection and processed for total RNA or total protein isolation^43^, using either TRIzol or spin-column RNA extraction, followed by treatment with DNA-Free kit.

### *NKX2-1-AS1* cloning and overexpression

The full-length *NKX2-1-AS1* (1,775 bp) was synthesized and cloned in the Nhe-BamH1 sites of the pCDNA3.1+ vector (GenScript). Overexpression of *NKX2-1-AS1* or empty vector control was performed in H441 or A549 cells using Lipofectamine LTX with Plus™ reagent (Life Technologies). RNA was isolated after 48 h and expression of *NKX2-1-AS1* and *NKX2-1* and other genes determined by RT-qPCR.

### Promoter-Luciferase activity studies

A 1.1 kb fragment upstream of the transcription start site of the human *CD274* gene was synthesized and cloned in the pGL3-basic plasmid (Promega) (*CD274*-Luc) (GenScript). A549 cells were transfected with various constructs using Lipofectamine 3000 (ThermoFisher); the total amount of DNA used in each transfection was maintained constant by completion with pCDNA3.1 + plasmid. −1kbCD274-Luc (4ug) or 0-Luc control were transfected in the presence of various concentrations of pCDNA3.1-*NKX2-1-AS1* vector (0, 0.5, 1, 2.5, 3, 5, 7 ug) alone or in combination with the pCDNA3.1-*NKX2-1* vector (2ug). pCMV-Green Renilla luciferase vector construct (ThermoFisher) was co-transfected to measure transfection efficiency. Dual-Luciferase Reporter assay system (Promega) was used to measure luciferase activity in a Biotex Synergy 2 instrument following the manufacturer’s protocol. Total protein concentration and Renilla luciferase activity were used for normalization. RT-qPCR analyses were performed on the same samples to assess level of overexpression of *NKX2-1* and *NKX2-1-AS1* and endogenous *CD274* using assays described in Supplementary Table S1.

### Western blots

Protein extracts were run on Nu-Page 4-12% Bis-Tris polyacrylamide gels (Invitrogen) and transferred to nitrocellulose membranes in an iBlot2 (Thermo Fisher Scientific). Blots were treated with Odyssey blocking buffer (LI-COR) at room temperature for 1 h and incubated with rabbit monoclonal TTF1 (*NKX2-1*) antibody (Abcam ab133737; 1:4000) and mouse monoclonal β-actin antibody (Sigma A54441; 1:4000) overnight at 4 °C overnight, and detected with anti-rabbit HRP conjugated secondary antibody (company catalog; 1:5000), and detected with anti-rabbit IgG DyLight 800 from (Cell Signaling Technology 5151S; 1:8000 in 1XTBST) and anti-mouse IgG DyLight 680 (Invitrogen 35518; 1:8000 in 1XTBST) and quantitated using a LI-COR imaging system. PD-L1 and PTPN1 (PTP1B) proteins were similarly analyzed using mouse PD-L1 antibody (Abcam ab210931; 1:4000) and rabbit PTP1B antibody (Cell Signaling Technology 5311S; 1:2000), respectively.

### Growth, proliferation and apoptosis assays

To determine the effect of *NKX2-1-AS1* levels on cell growth, H441 cells were transfected with the mix of three siRNAs or control as described above, cultured, trypsinized and counted at 0 h, 24 h, 48 h, and 72 h after seeding. Proliferation was determined with Click-iT® EdU Flow Cytometry Assay Kit (Invitrogen) coupled to Alexa Fluor®488 dye and analyzed by standard flow cytometry methods at the Boston University Flow Cytometry Core to determine the percentage of cells in each cell cycle phase. Apoptosis was determined using ApoDETECT™ (Invitrogen) that uses Annexin V-FITC and propidium iodide to detect cell surface exposure of phosphatidylserine in viable cells by flow cytometry following the manufacturer’s protocol.

### Wound healing, migration and invasion assays

We tested the effect of reduced *NKX2-1-AS1* levels on the wound healing behavior of H441 cell monolayers upon induced injury. Healing of a scratch in confluent H441 cells transfected with non-silencing control or *NKX2-1-AS1* siRNAs was analyzed using the analytical function in Adobe Photoshop CS4 Suite. Migration and invasion were determined using CytoSelect™ 24-well Cell Migration and Invasion Assay (8 µm, Fluorometric Format) (Cell Biolabs, Inc.). Cell migration analysis was performed 48 h and 72 h after *NKX2-1-AS1* siRNAs transfection using polycarbonate membrane inserts. After 24 h migrating cells were dissociated from the membrane and detected in a fluorescence reader using the CyQuant® GR Dye (Invitrogen). For invasion assays we used basement membrane-coated inserts following the same protocol.

### Microarray analysis

Affymetrix Human Gene 2.0 ST arrays were used to profile RNA (extracted using either TRIzol or spin columns) from H441 cells treated with the pool of three siRNAs targeting the lncRNA *NKX2-1-AS1* or non-silencing control (n = 3 per group). Arrays were normalized using the Robust Multiarray Average (RMA) with the affy R package (version 1.36.1) and an Entrez Gene-specific probeset mapping (version 16.0.0) from the Molecular and Behavioral Neuroscience Institute at the University of Michigan. Differential expression was assessed using the moderated (empirical Bayesian) *t* test implemented in the limma R package (version 3.14.4); specifically, simple linear models were created using lmFit, adjusting for RNA extraction protocol, followed by empirical Bayesian adjustment with eBayes. Correction for multiple hypothesis testing was accomplished using the Benjamini-Hochberg false discovery rate (FDR), performed across the genes that were expressed above the median value of at least one of the 12 microarrays. All analyses were performed using the R environment for statistical computing (version 2.15.1). Gene Set Enrichment Analysis (GSEA) (version 2.0.13) was used to perform pre-ranked analyses (default parameters with random seed 1234), using a list of all Entrez Gene identifiers ranked by the *t* statistic computed between *NKX2-1-AS1* knockdown and control, and the Entrez Gene versions of the Biocarta, KEGG, Reactome, Gene Ontology (GO), cytoband, and transcription factor and microRNA motif gene sets obtained from the Molecular Signatures Database (MSigDB), version 4.0. Detailed analyses are included in the Supplementary Materials.

### *NKX2-1-AS1* locus amplification analyses

Genomic DNA corresponding to the tumors described in Table 1 obtained from OriGene was used to determine the copy number of the *NKX2-1-AS1* locus using a custom designed SYBR Green Assay (Supplementary Table S1). To normalize we used a SYBR Green assay designed to amplify *LINE1* loci (Supplementary Table S1), a repetitive sequence with conserved copy number in most human normal and neoplastic cells^26,27^. Normal human iPSC DNA was used to generate the calibration curve.

### Chromatin Immunoprecipitation Assays (ChIP)

Analyses were performed with the MAGnify Chromatin Immunoprecipitation kit (ThermoFisher) according to the manufacturer’s protocol with minor modifications. Cells were trypsinized, washed with 1 x PBS and fixed with 1% formaldehyde in 1x PBS at room temperature with rotation for 10 min. Chromatin was sonicated to 300–500 bp fragments using Pico Bioruptor (Diagenode) for 12 cycles 30 sec on, 30 sec off. Lysate was centrifuged at 20,000 × g for 5 min at 4 °C to remove insoluble material. DNA content was measured in a NanoDrop and equal amount of soluble chromatin was used for ChIP assays. 5ug of anti-TTF1 antibody (*NKX2-1* protein)(Abcam, ab133737) and nonspecific rabbit IgG were added to sheared chromatin and incubated with rotation at 4 °C overnight. ChIPs were analyzed by qPCR using 2X Fast SYBR master mix (ThermoFisher) and promoter-specific assays (Supplementary Table S1).

### RNA Immunoprecipitation Assays (RIP)

Analyses were performed with the Nuclear RNA-Binding Protein Immunoprecipitation Kit (Millipore) according to manufacturer’s protocol with minor modifications. Cells were grown in 150 mm culture dish and allowed to reach 90% confluency. Cells were crosslinked using 1% formaldehyde in media, and washed with 1x PBS supplemented with protease and RNAse inhibitors. Nuclear extracts were isolated and sonicated to 100–300 bases fragments using Pico Bioruptor (Diagenode) for 10 cycles (30 sec on and 30 sec off). Lysates were centrifuged at 20,000 × g for 10 min at 4 °C to remove insoluble material. RIP incubations were performed overnight at 4 °C with rotation using 5ug or 10 ug of anti-TTF1 (*NKX2-1*) antibody (Abcam, ab133737) or nonspecific rabbit IgG. One aliquot was saved for input. RNA purification was performed using RNeasy kit (Qiagen). RT was performed using SensiScript kit (Qiagen) and qPCR was performed using 2X Fast Taqman master mix (ThermoFisher) and assays to detect *NKX2-1-AS1* lncRNA and *NKX2-1* mRNA as control (Supplementary Table S1).

### Statistical Analysis

Data were obtained from at least three independent experiments (n ≥ 3) and presented as mean ± SEM unless other stated in the Figure legend. The significance of differences was calculated using parametric *t*-test for two-group unpaired comparisons, two-tails; non-parametric t-tests were used to analyze the RIP and ChIP assays. *p* < 0.05 was considered statistically significant. For tumor samples, we used the Wilcoxon Mann Whitney test to analyze expression values of *NKX2-1* and *NKX2-1-AS1* in adenocarcinoma (n = 8) relative to their corresponding non-tumor control versus squamous cell tumors (n = 8) relative to their corresponding non-tumor controls. All statistical analyses were performed in GraphPad Prism 7.0c.

## Electronic supplementary material

Supplementary Material

## Data Availability

The datasets generated and analyzed during this study are available in the Gene Expression Omnibus (GEO) repository, Series GSE104691. This work was partially supported by R01 HL127426 (MIR) and CTSA grant UL1-TR000157.
