# Supplementary material for: NKX2-1-AS1 negatively regulates CD274/PD-L1, cell-cell interaction genes, and limits human lung carcinoma cell migration
Source: Sci Rep. 2018 Sep 26;8:14418. doi: 10.1038/s41598-018-32793-5 (PMC6158174; doi:10.1038/s41598-018-32793-5)
Supplement: Supplementary file 1 — Supplementary Material [file 41598_2018_32793_MOESM1_ESM.pdf]

NKX2-1-AS1 negatively regulates CD274/PD-L1, cell-cell interaction genes and limits human lung carcinoma cell migration

Hasmeena Kathuria, Guetchyn Millien, Liam McNally, Adam C. Gower, Jean-Bosco Tagne, Yuxia Cao, and Maria I. Ramirez.

Supplementary Materials

Supplementary Figures S1-S9

Supplementary Table S1

Microarray Analysis Report

Supplementary Figure S1

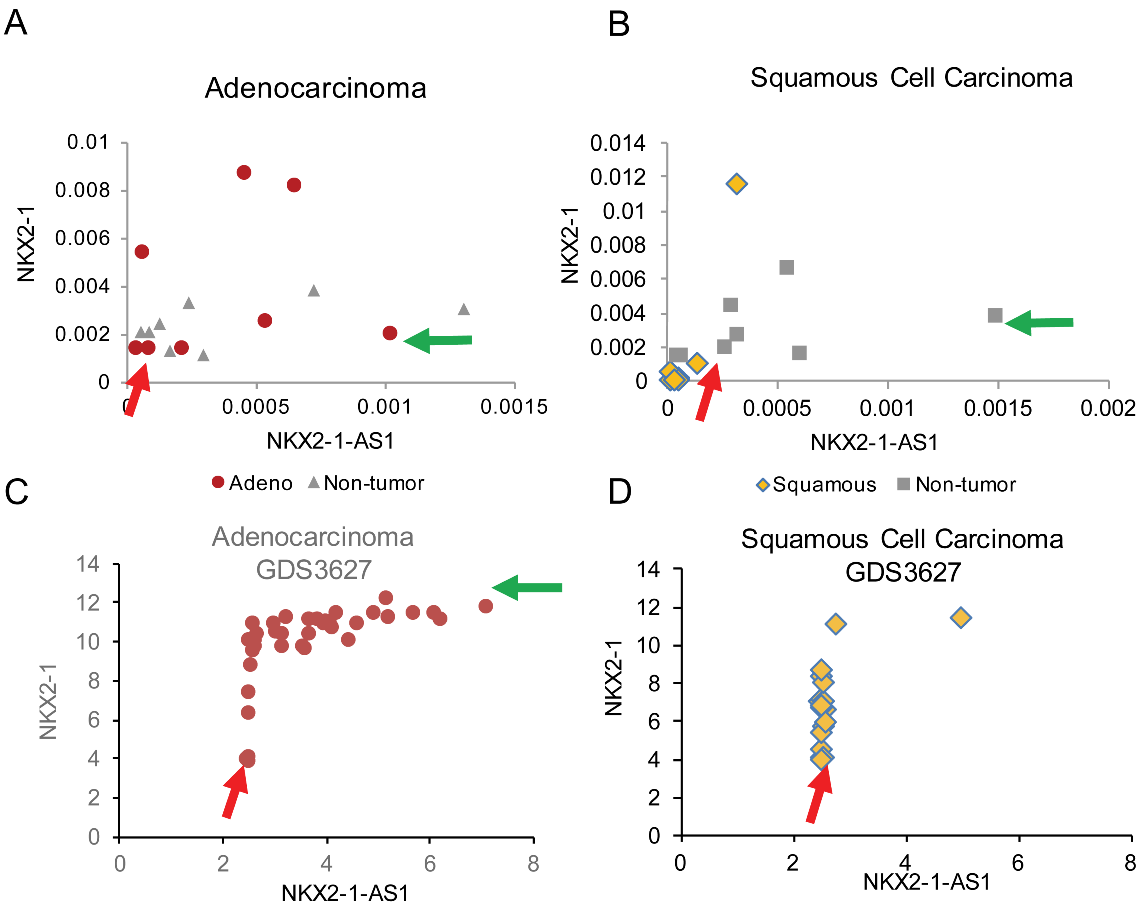

Supplementary Figure S1: Correlation plots of the expression of NKX2-1-AS1 and NKX2-1. Expression (A) determined by qPCR in the adenocarcinomas (red, n=8), and the corresponding non-tumor control (gray, n=8); and (B) in squamous cell carcinomas (yellow, n=8), and corresponding non-tumor control (gray, n=8) in in this study. (C) Adenocarcinomas in GEO dataset GDS3627 (n=40). (D) Squamous cell carcinomas in GDS3627 (n=18). Expression values in panels C and D are in log2 scale. Red arrows point to tumors with constant levels of NKX2-1-AS1 and variable NKX2-1. Green arrows point to samples with variable levels of NKX2-1-AS1 and constant levels of NKX2-1, portraying the variability in the relative expression of NKX2-1 and NKX2-1-AS1 in these tumors.

Supplementary Figure S2

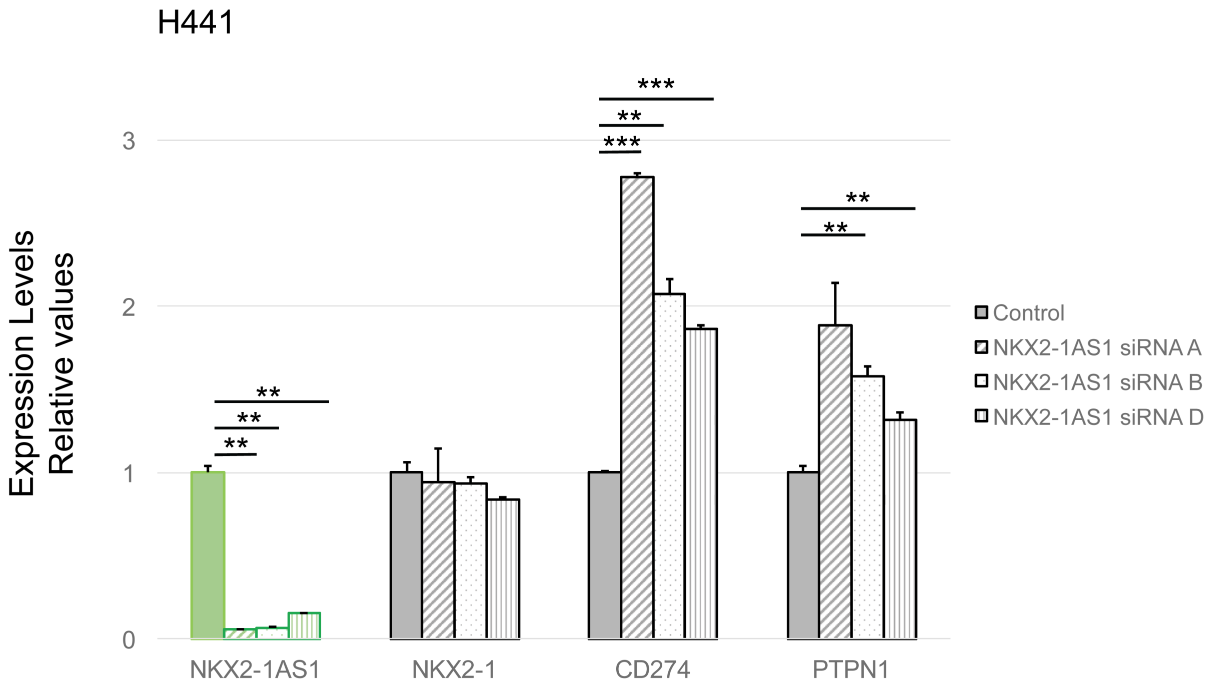

Supplementary Figure S2: Individual siRNAs A, B and D were able to down-regulate NKX2-1-AS1 expression by >80% in H441 cells, similar to the combination of all three siRNAs used in the experiments in this study, which was used to reduce non-specific effects of individual siRNAs. NKX2-1 mRNA expression was not significantly changed by NKX2-1-AS1 knockdown with any of the individual siRNAs. Treatment with individual siRNAs (A, B, or D) significantly increased expression of CD274, and treatment with B or D significantly increased expression of PTPN1, both genes identified as downstream of NKX2-1-AS1 in this study. n=3; ** $p < 0.01$; ***$p < 0.001$.

Supplementary Figure S3

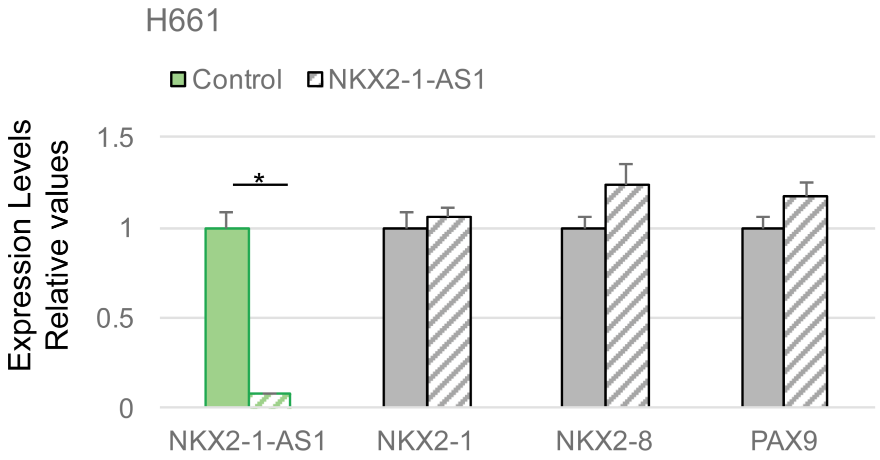

Supplementary Figure S3: Knockdown of NKX2-1-AS1 in H661 using the mix of 3 siRNAs had no effect on the expression of neighboring genes. As was observed in H441 cells, downregulation of NKX2-1-AS1 > 90% at 48h did not significantly change the levels of NKX2-1 and NKX2-8. Unlike in H441 cells, however, down-regulation of NKX2-1-AS1 did not affect PAX9 levels. n=3; * $p<0.05$.

Supplementary Figure S4

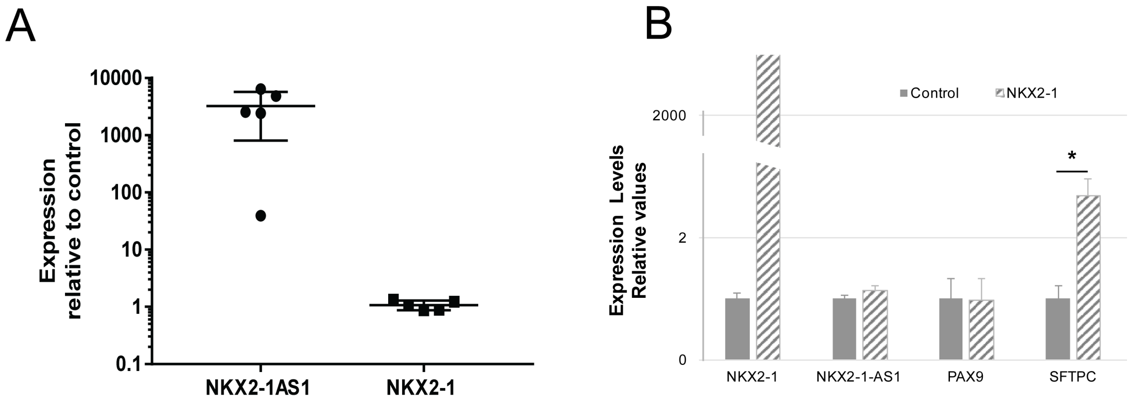

Supplementary Figure S4 (A) Overexpression of NKX2-1-AS1 in H441 cells showed no effect on the levels of expression of NKX2-1 (n=3, *p<0.05). (B) Overexpression of NKX2-1 in H441 cells did not affect expression of NKX2-1-AS1 or PAX9 but increased transcription of its known target gene SFTPC (n=3; *p < 0.05).

Supplementary Figure S5

A

B

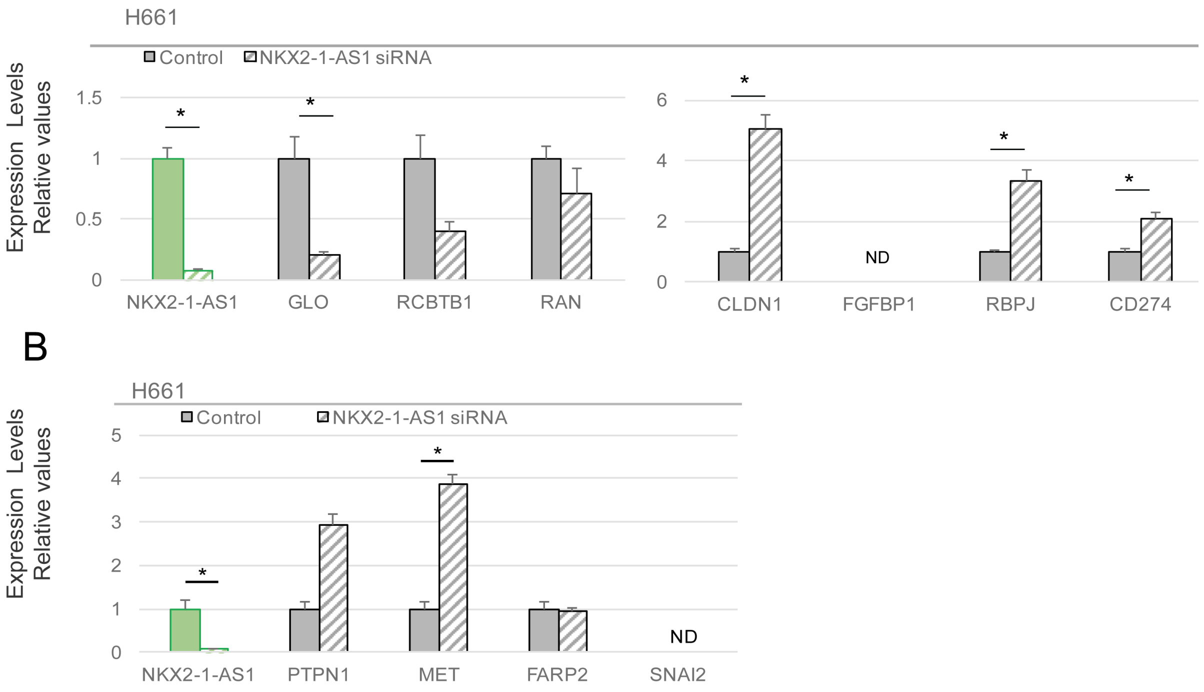

Supplementary Figure S5: NKX2-1-AS1 knockdown altered gene expression in the same manner in H661 and H441 cells. (A) qPCR validation of down-regulated and up-regulated genes in NKX2-1-AS1 knockdown H661 cells at 48h after treatment (n=3, * p < 0.05, **p < 0.01). (B) Real time PCR validation of adherens junctions related genes up-regulated by NKX2-1-AS1 knockdown in H661 cells (n=3; *p <0.05; ND = not detected).

Supplementary Figure S6

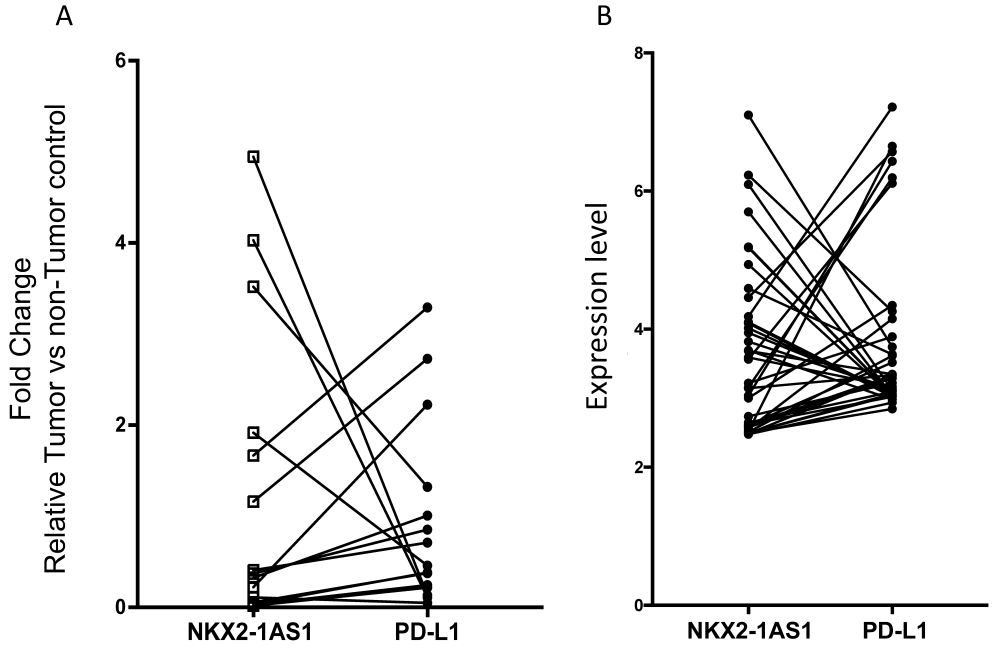

Supplementary Figure S6: (A) NKX2-1-AS1 and CD274 (PD-L1) mRNA levels exhibited an inverse pattern of regulation in the human NSCLC specimens tested in this work. An increase in NKX2-1-AS1 expression in tumors compared to its corresponding non-tumor control mainly corresponds to a downregulation of CD274 (PD-L1) expression in tumors (determined by qPCR, n=16). (B) An inverse trend between NKX2-1-AS1 and CD274 (PD-L1) expression in lung tumors is observed in the publicly available GEO dataset GDS3627 (expression values in log2 scale).

Supplementary Figure S7

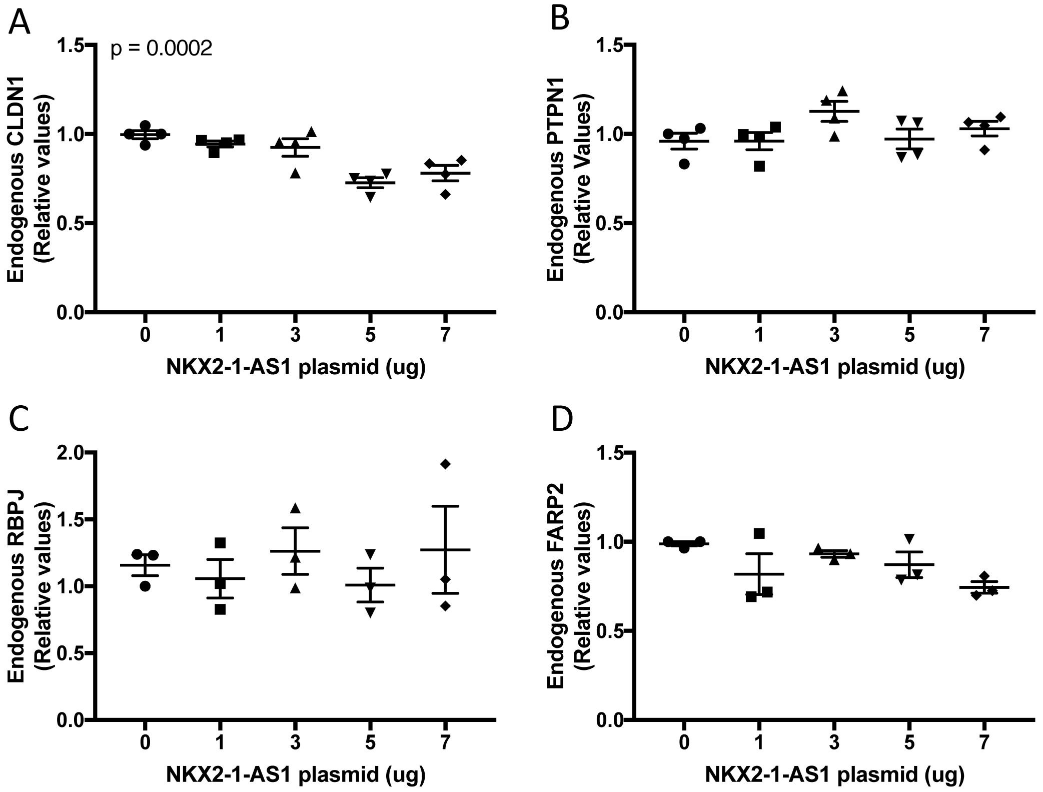

Supplementary Figure S7: Overexpression of NKX2-1-AS1 in A549 cells showed (A) decreased expression of endogenous CLDN1 (n=4; ANOVA p=0.0002), but no changes in (B) PTPN1 (n=4), (C) RBPJ (n=3) and (D) FARP2 (n=3).

Supplementary Figure S8

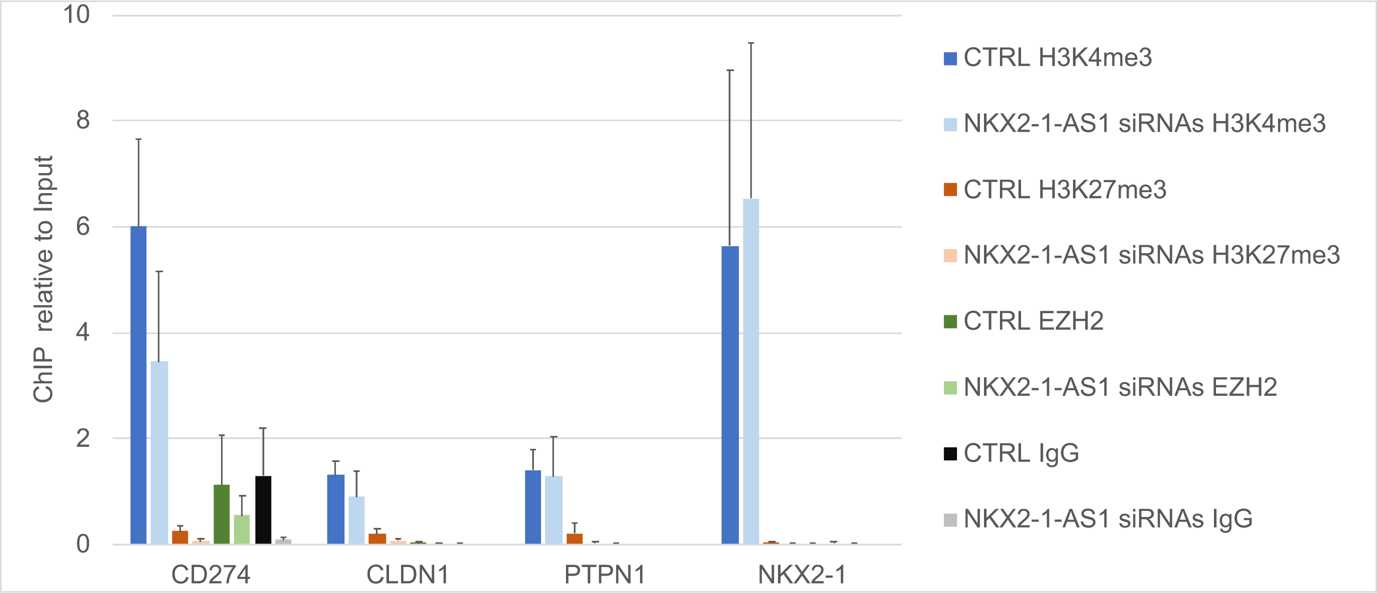

Supplementary Figure S8: ChIP-qPCR analysis of histone methylation marks H3K4me3 and H3K27me3, and binding of the histone methyl transferase EZH2 compared to IgG negative control on the promoter regions of CD274, CLDN1, PTPN1 and NKX2-1 in H441 cells transfected with NKX2-1-AS1 siRNAs or non-silencing control (n=3).

Supplementary Figure S9

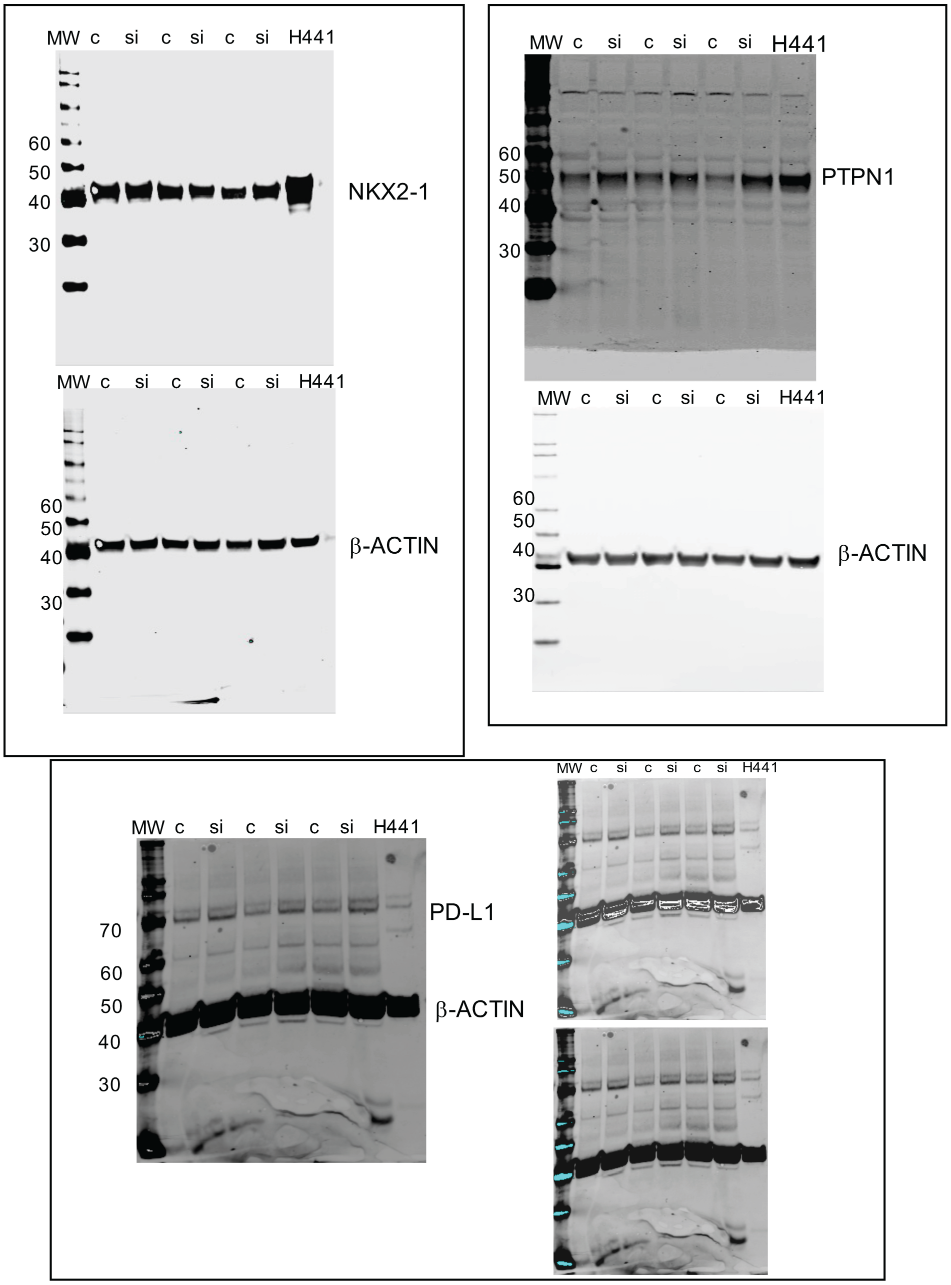

Supplementary Figure S9: Full Western blot images for Figure 3D and Figure 4E.

Supplementary Table S1. Assays and oligonucleotide sequences.

NAME	Assay ID number/sequence
**NKX2-1 AS1**	AJHSOEQ Custom Thermo Fisher Target chr14:36988483-36992221 GRCh38 139bp and Hs04408121_m1
**NKX2-1 both variants**	Hs00968940_m1
**NKX2-1 single variant**	Hs03968940_m1
**MBIP**	Hs00968947_m1
**SFTPC**	Hs00161628_m1
**SLC25A21**	Hs00229049_m1
**PTPN1**	Hs00942477_m1
**MET**	Hs00179845_m1
**FARP2**	Hs00919572_m1
**SNAI2**	Hs00161904_m1
**CLDN1**	Hs00221623_m1
**FGFBP1**	Hs01921428_s1
**RBPJ**	Hs00794653_m1
**CD274**	Hs00204257_m1
**NKX2-1AS1 Copy #1**	AJ6RN26 Custom Thermo Fisher Target chr14:36990354-36990848 (GRCh38) 96bp
**LINE1**	Line3_CC39RNO Custom Thermo Fisher Target 1-210 bp
**NKX2-8**	Hs00180876_m1
**b-ACTIN**	Hs99999903_m1
**TGFBI**	Hs00171257_m1
**SFTA3**	Hs01393841_m1
**NGSP1**	5'-GATTACGCCAAGCTTGTCGATGAGTCCAAAGCACACGACTCCG-3'
**siRNA sequences**	siRNA Name: NKX2-1AS1 siRNA A<br>SENSE: AAGGAAGAGUUGGGUGAAUtt<br>ANTISENSE: AUUCACCCAACUCUUCCUUtc<br><br>siRNA Name: NKX2-1AS1 siRNA B<br>SENSE: ACUGAGCAUUGAUCACUGUtt<br>ANTISENSE: ACAGUGAUCAAUGCUCAGUtc<br><br>siRNA Name: NKX2-1AS1 siRNA D<br>SENSE: CUGCAGUUCUCGAGGAAACtt<br>ANTISENSE: GUUUCCUCGAGAACUGCAGtc<br>Silencer® Select negative control no. 1 siRNA (4390843)
**CD274 promoter**	Qiagen, GPH008505(-)01A
**PTPN1 promoter**	Qiagen, GPH1023457(-)01A
**CLDN1 promoter**	Qiagen, GPH1012902(-)01A

Microarray analysis report

Summary of microarray samples and experimental design

This study is comprised of two independent experiments, each containing 6 Human Gene 2.0 ST arrays profiling H441 lung adenocarcinoma cells treated with control siRNA or a pool of three siRNAs (A, B and D) targeting the lncRNA *NKX2-1-AS1* (n=3 per group in each experiment). Trizol RNA extraction was used to prepare samples for the first experiment (Experiment 1), while spin-column RNA extraction was used to prepare samples for the repeat of the first experiment (Experiment 2).

Normalization and quality assessment

All 12 arrays were normalized together using the Robust Multiarray Average (RMA) algorithm and a CDF (Chip Definition File) that maps the probes on the array to unique Entrez Gene identifiers. The result is a matrix in which each row corresponds to an Entrez Gene ID and each column corresponds to a sample. The expression values are log2-transformed by default.
The technical quality of the arrays was assessed by two quality metrics: Relative Log Expression (RLE) and Normalized Unscaled Standard Error (NUSE). For each sample, median RLE values > 0.1 or NUSE values > 1.05 are considered out of the usual limits, although RLE is the quality metric most strongly associated with technical quality. All arrays had median RLE and NUSE values well within these limits.

Positive control gene expression

The expression of several constitutively expressed Y-linked genes (*DDX3Y*, *KDM5D*, *RPS4Y1*, *USP9Y*, and *UTY*) was assessed to estimate the dynamic range of the array, as these genes will serve as strong positive or negative expression controls in males and females, respectively. In both experiments the expression of *XIST* was very low (~1-2 log2 units) in all samples, and although the expression of most Y-linked genes was also relatively low (~2-4 log2 units), the *DDX3Y* had moderate expression (~5 log2 units) in all samples across both experiments. These findings are in agreement with the fact that the H441 cell line was derived from a male subject and contains a Y chromosome (http://www.atcc.org/products/all/HTB-174.aspx). As other housekeeping genes had high expression, there appears to be good dynamic range to discriminate true positive and negative controls in this experiment.

The expression of *NKX2-1-AS1* was decreased ~1.2-fold in the knockdown group versus the control group in both experiments (reduction to 83% of original levels in Experiment 1 and 81% of original levels in Experiment 2).

Principal Component Analysis (PCA)

Following the initial QC analysis, Principal Component Analysis (PCA) was performed. PCA is a mathematical transform that collapses the variance between samples across a set of large set of variables (here, all ~25,000 genes on the array) into a much smaller set of variables called Principal Components (PCs). These "meta-variables" are arranged such that PC1 explains the most variance in the data, followed by PC2, etc.

PCA was performed using all genes across all samples, and a plot was made of PC2 vs. PC1 for both experiments independently and then combined.
The plots can also be downloaded at:
[2014-08-08_Ramirez_combined_PCA.pdf]

Figure 1. PCA analysis of combined samples from Experiments 1 and 2, unadjusted for experimental protocol.

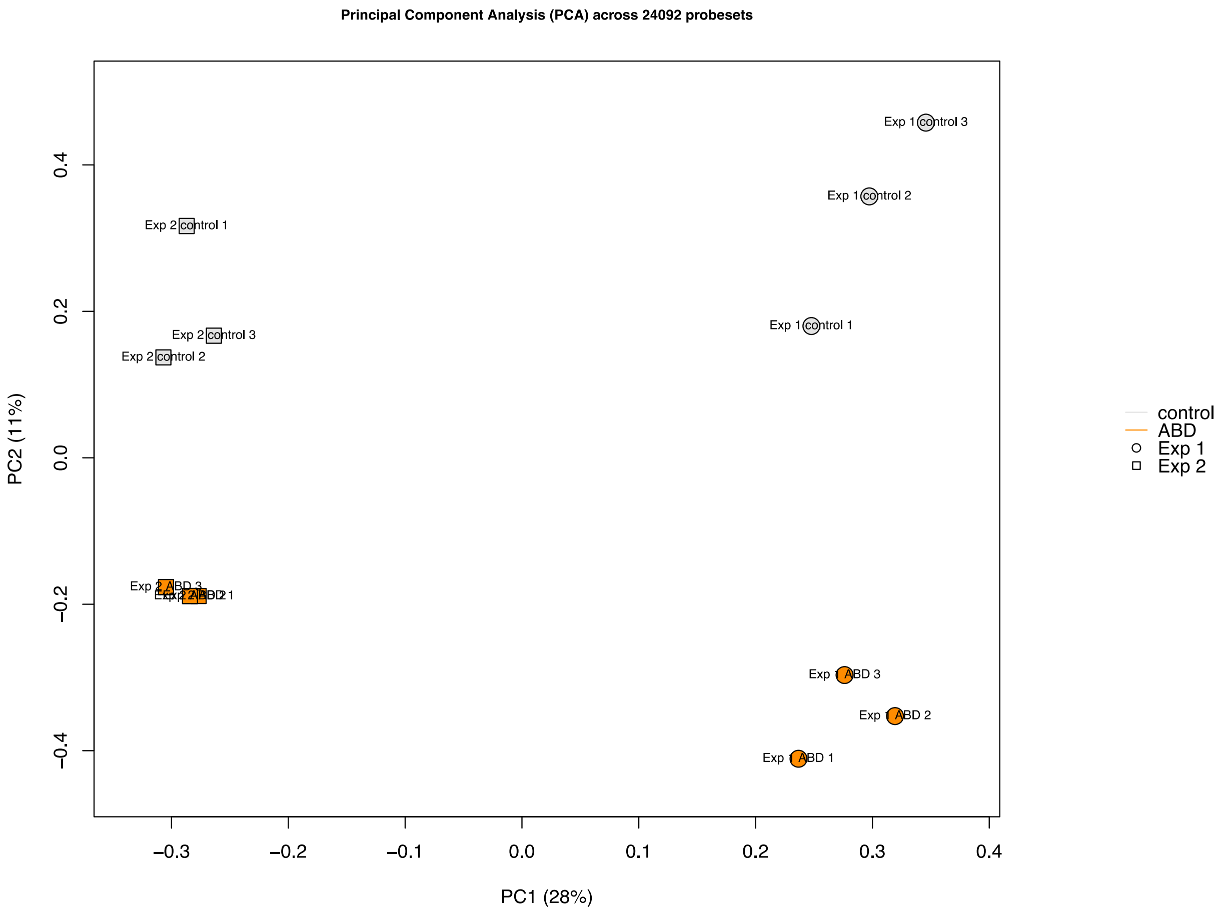

Samples shown in Figure 1 separate on the PCA plot by experiment along the PC1 axis, which explains 28% of variance in the experiment. This suggests that either the RNA extraction protocol or batch effect is the major cause of variance between the two experiments. However, in both experimental groups, control samples separate well from the NKX2-1-AS1 knockdown samples along the PC2 axis, which explains 11% of the variance in the experiment, suggesting that the siNKX2-1-AS1 treatment caused the most variance within each experiment.

Figure 2. PCA analysis of combined samples from Experiments 1 and 2, adjusted for experimental protocol.

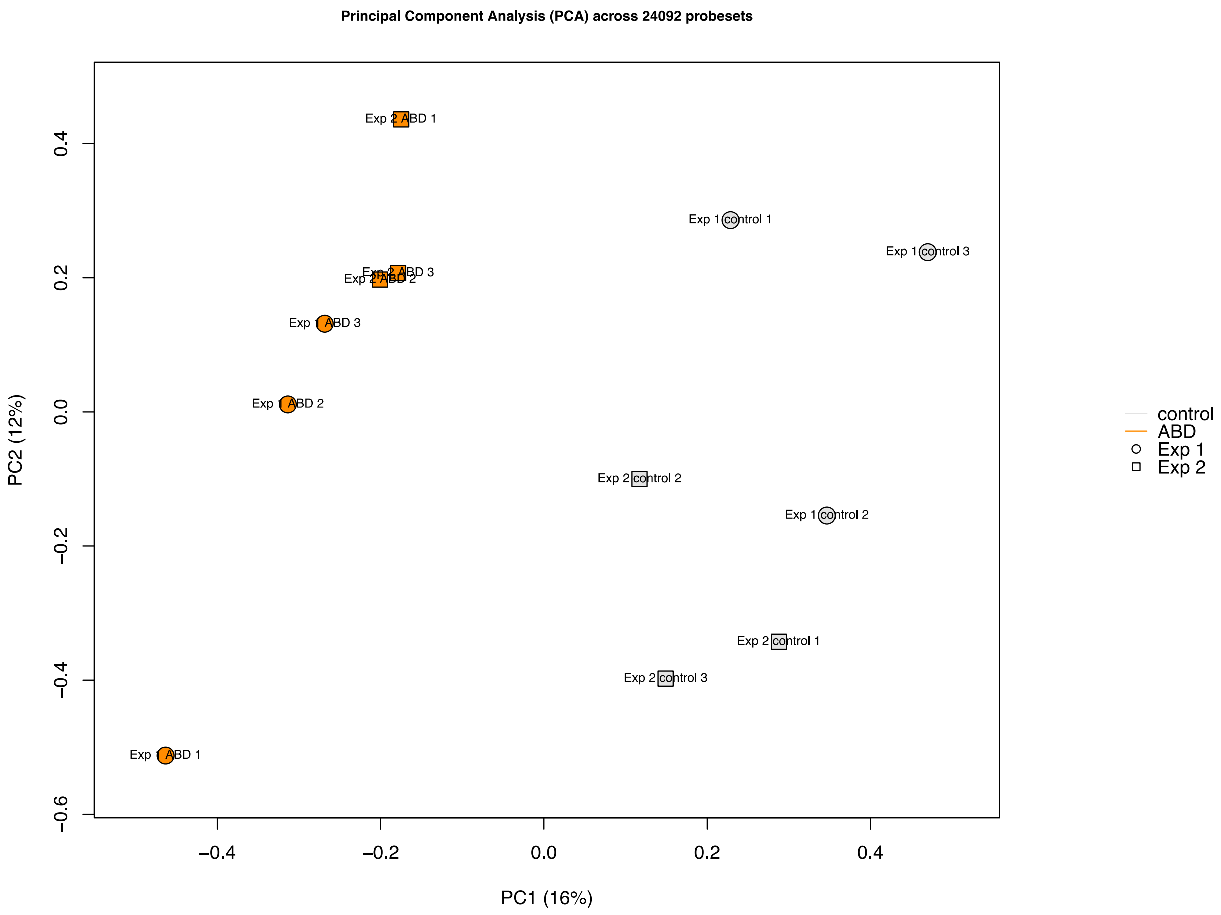

Figure 2 shows the adjustment for experimental protocol leading to a distinct separation between all of the control and all of the NKX2-1-AS1 knockdown samples from both experiments along the PC1 axis, which is responsible for 16% of variance in the experiment. However, in both the control samples and the ABD clusters, the distinct experimental groups separate from each other. This suggests that after adjusting for experimental protocols, the treatment becomes the most important cause in variance across all samples, and the separation between the experimental groups may potentially be explained by the two different RNA extraction methods.

Moderated t tests: NKX2-1-AS1 knockdown versus control

To identify genes whose expression changed with respect to treatment after adjusting for experimental protocol, a linear model was created of the form

```
expression ~ protocol + treatment
```

where '~' means 'is a function of', and *protocol* and *treatment* are treated as categorical variables. The *treatment* effect therefore measures whether a given gene changes expression between siRNA groups after correcting for any technical effects introduced by the two experiments.

A *t* test was then performed for the treatment term in the model to determine its significance. A "moderated" *t* test was used, which is a Bayesian analysis that does not test each gene independently, but rather, leverages information from all of the genes on the array to increase statistical power over a standard two-sample Student *t* test. It is especially helpful when sample sizes are small.

Benjamini-Hochberg False Discovery Rate (FDR) correction was then applied to obtain FDR-corrected p values ('q' values), which represent the probability that a given result is a false positive based on the distribution of all p values on the array. Corrected/adjusted p values such as the FDR q are the best measure of significance for a given test when many hypotheses (e.g., ~25,000 genes) are tested at once. In addition, the FDR q value was also recomputed after removing probesets that were not expressed above the array-wise median value of at least one array. Probesets with low overall expression are more strongly affected by random technical variation and more likely to produce false positive results.

Table 1 below shows the number of genes with a *p* value below various thresholds, as well as the number of genes expected by chance at each threshold. Similarly, the table below also shows the number of genes with a q value below various thresholds, either with or without expression filtering.

Table 1. Summary of significant p and FDR q values between NKX2-1-AS1 and control siRNA.

p threshold	Expected	ABD vs control	q threshold	all genes	expression filter
0.05	1205	2840	0.25	1283	1691
0.01	241	1094	0.1	561	736
0.005	120	776	0.05	341	458
0.001	24	400	0.01	95	140

As was expected, when combining all samples from the two experiments and adjusting for experimental protocols, there are considerable differential gene expressions changes between the cells treated with NKX2-1-AS1 siRNA and control siRNA. This is further reflected in the large number of genes that pass FDR q correction.

Summary of statistical analyses
The analyses are summarized in the following Excel file:
2014-08-08_Ramirez_combined_analysis.xlsx

This file contains:

- annotation for each gene
- signed linear fold changes for *NKX2-1-AS1* knockdown vs control
  (e.g., +2 = 2-fold higher in *NKX2-1-AS1* knockdown than in control; -2 = 2-fold lower in *NKX2-1-AS1* knockdown than in control)
- moderated t statistics, p values and FDR q values
- log2 (expression) across all samples, laid over a colored representation (heatmap), scaled so that red and blue indicate expression values ≥ 2 standard deviations above and below, respectively, the row-wise mean (white) computed across all samples

The rows are sorted in ascending order by moderated t statistic.

Gene Set Enrichment Analysis (GSEA)

Gene Set Enrichment Analysis (GSEA) was then used to identify biological terms, pathways and processes that were overrepresented among the genes that were up- or down-regulated with respect to treatment group. Briefly, the idea behind Gene Set Enrichment Analysis (GSEA) is that it determines whether, on the whole, a given set of genes tends to be more up-regulated or down-regulated in a given comparison. If the set of genes is related to some biological pathway or process, then a significant GSEA result for that gene set suggests that that pathway/process is relevant to the experimental comparison at hand.

GSEA requires two inputs:

1. *A list of all genes on a platform that is ranked according to some metric.*
   Here, the Entrez Gene IDs of all genes on the Human Gene 2.0 ST array have been ranked by the moderated t statistics computed for the treatment effect in the linear model after adjusting for experimental protocol.
2. *A collection of gene sets to test.*
   Here, a collection of gene sets was obtained from the publicly available Molecular Signatures Database, or MSigDB (http://www.broadinstitute.org/gsea/msigdb/index.jsp) (version 4.0), which is maintained by the same group that developed GSEA. Gene sets corresponding to biological pathways, locations, or functions are derived from the following public databases: Kyoto Encyclopedia of Genes and Genomes (KEGG), Gene Ontology (GO), Biocarta, and Reactome. Additionally, sets of genes were also included that are computationally predicted to contain transcription factor or microRNA binding sites in their promoters or 3' UTRs, respectively (note that these motifs may or may not be associated with known regulators), or which are located within a given chromosomal location.

For each ranked list, the GSEA algorithm tests each gene set in turn to determine whether its members are distributed nonrandomly within the ranked list. It then assigns a p value to each gene set based on how skewed the distribution of the gene set is towards the up- or down-regulated end of the ranked list (weighted by the ranking metric, so that the genes at the extreme end of the ranked list have more importance in computing the p value).

An Excel file summarizing the GSEA results is available at:
2014-08-08_Ramirez_combined_GSEA.xlsx

The columns in the file are as follows:

A. Group: the category of the gene set
B. Gene Set Name: the name provided by MSigDB
C. Link: A link to the "card" with more information about each gene set at MSigDB
D. Gene Set Size: The number of genes in the gene set that overlap with the genes in the ranked list
E. ES: The Enrichment Score, or skewness of each gene set.
   A positive ES means the genes in the set are predominantly up-regulated with respect to the reference group, and a negative ES means the genes in the set are predominantly down-regulated with respect to the reference group.
F. NES: The Normalized Enrichment Score (ES normalized to gene set size), used to compute significance of each gene set
G. Nominal p value: The p value computed from the NES; NOTE: this can be equal to 0, since it is computed by permutation
H. FDR q-value: the FDR-corrected p value; FDR q < 0.25 is in ***bold italics***

The rows have been sorted in ascending order by NES (column F) and filtered to show only those for which FDR q < 0.25 (column H).

The results can be browsed via the following link:
NKX2-1-AS1_knockdown_vs_control/

This link allows for detailed exploration of the genes in the gene sets with FDR q < 0.25, including:

- an enrichment plot showing the position of each gene in the gene set along the ranked gene list (with red and blue indicating positive and negative t statistics), as well as the running Enrichment Score (ES) (green line), the maximal distance from the x-axis indicating the final ES
- annotation of each gene in the gene set, including a link to the Entrez Gene page
- annotation of the genes with Core Enrichment (the "leading edge" of the gene set), i.e., those genes that have the greatest impact on the enrichment score for the gene set
- a link to a tab-delimited text version of the table (which is given an .xls extension to open automatically in Microsoft Excel)

A ZIP archive of the GSEA run folder can also be downloaded via the following link:
2014-08-08_Ramirez_combined_GSEA.zip

Funding Source
Please acknowledge CTSA grant UL1-TR000157 in any publications resulting from this analysis.

Methods

Microarray analysis

Affymetrix GeneChip Human Gene 2.0 ST CEL files were normalized to produce gene-level expression values using the implementation of the Robust Multiarray Average (RMA) [1] in the *affy* package (version 1.36.1) [2] included within in the Bioconductor software suite (version 2.12) [3] and an Entrez Gene-specific probeset mapping (version 16.0.0) from the Molecular and Behavioral Neuroscience Institute (Brainarray) at the University of Michigan [4,5]. Array quality was assessed by computing Relative Log Expression (RLE) and Normalized Unscaled Standard Error (NUSE) using the *affyPLM* Bioconductor package (version 1.34.0) [6]. Array quality was assessed by computing Relative Log Expression (RLE) and Normalized Unscaled Standard Error (NUSE) using the *affyPLM* Bioconductor package (version 1.34.0) [6]. Principal Component Analysis (PCA) was performed using the *prcomp* R function with expression values that had been normalized across all samples to a mean of zero and a standard deviation of one. Pairwise differential microRNA expression was assessed using the moderated (empirical Bayesian) t test implemented in the *limma* package (version 3.14.4) (i.e., creating simple linear models with *lmFit*, followed by empirical Bayesian adjustment with *eBayes*). Correction for multiple hypothesis testing was accomplished using the Benjamini-Hochberg false discovery rate (FDR) [7]. All statistical analyses were performed using the R environment for statistical computing (version 2.15.1).

Gene Set Enrichment Analysis (GSEA)

GSEA (version 2.0.13) [8] was used to identify biological terms, pathways and processes that were coordinately up- or down-regulated within each pairwise comparison. The Entrez Gene identifiers of the human homologs of the genes interrogated by the array were ranked according to the moderated *t* statistic computed for the treatment effect in a linear model adjusting for experimental protocol. This ranked list was then used to perform a pre-ranked GSEA analysis (default parameters with random seed 1234) using the Entrez Gene versions of the Biocarta, KEGG, Reactome, Gene Ontology (GO), cytoband, and transcription factor and microRNA motif gene sets obtained from the Molecular Signatures Database (MSigDB), version 4.0 [9].

References

1. Irizarry et al Biostatistics 2003, PubMed ID 12925520

2. Gautier et al Bioinformatics 2004, PubMed ID 14960456

3. Gentleman et al Genome Biology 2004, PubMed ID 15461798

4. Dai et al Nucleic Acids Res 2003, PubMed ID 16284200

5. http://brainarray.mbni.med.umich.edu/Brainarray/Database/CustomCDF

6. Brettschneider J, Collin F, Bolstad BM, and Speed TP. Quality Assessment for Short Oligonucleotide Microarray Data. Technometrics 2008; 50(3):241. DOI 10.1198/004017008000000334

7. Benjamini Y, Hochberg Y (1995). Controlling the false discovery rate: a practical and powerful approach to multiple testing. J Roy Statist Soc Ser B, 57(1):289-300.

8. Subramanian et al PNAS 2005, PubMed ID 16199517

9. Subramanian et al Bioinformatics 2007, PubMed ID 17644558
